# Extra virgin olive oil improved body weight and insulin sensitivity in high fat diet-induced obese LDLr−/−.Leiden mice without attenuation of steatohepatitis

**DOI:** 10.1038/s41598-021-87761-3

**Published:** 2021-04-15

**Authors:** Leticia Álvarez-Amor, Amparo Luque Sierra, Antonio Cárdenas, Lucía López-Bermudo, Javier López-Beas, Eloísa Andújar, Mónica Pérez-Alegre, Rocío Gallego-Durán, Lourdes M. Varela, Alejandro Martin-Montalvo, Genoveva Berná, Anabel Rojas, Mª José Robles-Frías, Abdelkrim Hmadcha, Manuel Romero-Gómez, Robert Kleemann, Franz Martín

**Affiliations:** 1 Andalusian Center of Molecular Biology and Regenerative Medicine-CABIMER, University Pablo Olavide-University of Seville-CSIC, Seville, Spain; 2 Biomedical Research Network On Diabetes and Related Metabolic Diseases-CIBERDEM, Instituto de Salud Carlos III, Madrid, Spain; 3 Hospital Universitario Virgen del Rocío de Sevilla, Instituto de Biomedicina de Sevilla, Universidad de Sevilla, Sevilla, Spain; 4 Biomedical Research Network On Hepatic and Digestive Diseases-CIBEREHD, Instituto de Salud Carlos III, Madrid, Spain; 5 Compared Pathology Unit, Hospital Universitario Virgen del Rocío-Intituto de Biomedicina de Sevilla Biobank Node, Andalucia Health Public System Biobank, Sevilla, Spain; 6 Department of Metabolic Health Research, Netherlands Organisation for Applied Scientific Research-TNO, 2333 CK Leiden, The Netherlands; 7 Department of Vascular Surgery, Leiden University Medical Centre, Leiden, 2333 ZA The Netherlands

**Keywords:** Type 2 diabetes, Non-alcoholic steatohepatitis

## Abstract

Dietary fatty acids play a role in the pathogenesis of obesity-associated non-alcoholic fatty liver disease (NAFLD), which is associated with insulin resistance (IR). Fatty acid composition is critical for IR and subsequent NAFLD development. Extra-virgin olive oil (EVOO) is the main source of monounsaturated fatty acids (MUFA) in Mediterranean diets. This study examined whether EVOO-containing high fat diets may prevent diet-induced NAFLD using *Ldlr−/−.* Leiden mice. In female *Ldlr−/−.*Leiden mice, the effects of the following high fat diets (HFDs) were examined: a lard-based HFD (HFD-L); an EVOO-based HFD (HFD-EVOO); a phenolic compounds-rich EVOO HFD (HFD-OL). We studied changes in body weight (BW), lipid profile, transaminases, glucose homeostasis, liver pathology and transcriptome. Both EVOO diets reduced body weight (BW) and improved insulin sensitivity. The EVOOs did not improve transaminase values and increased LDL-cholesterol and liver collagen content. EVOOs and HFD-L groups had comparable liver steatosis. The profibrotic effects were substantiated by an up-regulation of gene transcripts related to glutathione metabolism, chemokine signaling and NF-kappa-B activation and down-regulation of genes relevant for fatty acid metabolism. Collectivelly, EVOO intake improved weight gain and insulin sensitivity but not liver inflammation and fibrosis, which was supported by changes in hepatic genes expression.

## Introduction

The incidence of metabolic diseases has been growing worldwide, together with overweight and obesity^1^. NAFLD is thought to be the hepatic manifestation of the metabolic syndrome (SM)^2^ and has become the most common cause of chronic liver disease, representing a risk factor for cirrhosis, hepatocellular carcinoma and liver transplantation^3^. NAFLD is characterized by a disturbed fatty-acid metabolism and hepatic lipid accumulation. However, the pathogenesis of NAFLD is not fully understood. NAFLD development is thought to involve a genetic component as well as multiple simultaneous insults (“multiple hits hypothesis”). These hits include IR, inflammation, oxidative stress and nutritional factors^4^.

Dietary habits are one of the most important contributing factors to the development and progression of IR and NAFLD^5^. Hence, dietary strategies may also constitute effective treatments to attenuate IR and associated NAFLD development. Dietary fats are of interest because not only the total amount of fat consumed should be considered but also the type and quality of fats^6^ the more so because bioactive lipids exert specific biological functions.

High caloric diets, particularly those rich in trans fats, saturated fats, cholesterol, and fructose-sweetened drinks, can increase IR and visceral adiposity and are thought to stimulate lipid accumulation in the liver, as well as, progression of steatosis to nonalcoholic steatohepatitis (NASH)^5^. Indeed, experimental studies showed that diets rich in omega-3 polyunsaturated fatty acids (PUFA) can alleviate IR, intrahepatic triglyceride content and improve steatohepatitis^7^. A systematic review of clinical trials suggests that n-3 PUFA supplementation may be effective in the early stages of NAFLD, but not in patients with more severe NAFLD or NASH^8^. In case of MUFA, it is thought that MUFA could prevent NAFLD development^9^ and may reduce hepatic steatosis involving specific biochemical pathways^10^. However, other studies have drawn opposite conclusions, because MUFA consumption was positively associated with a higher fatty liver index^11^. Most of the studies that observe a negative relationship of MUFA consumption with NAFLD are cross-sectional studies in which the origin of MUFA is not considered. In a human study in which EVOO was used as a source of MUFA to treat type 2 diabetes (T2D) patients, a reduction of liver fat (39%) was observed^12^. In C57BL/6J mice pretreated with lard-based HFD, replacement of lard by EVOO reduced HFD-induced liver damage, paralleled by an anti-inflammatory effect in adipose tissue and modifications in liver lipid composition and hepatic signaling pathways^13^. However, recently it has been shown that in a female C57BL/6J saturated fat/fructose /cholesterol-rich diet-NAFLD model, the exchange of saturated fat for EVOO did not avoid the progression of NAFLD^14^.

In the past several years, the *Ldlr−/−* background has been frequently used as a model to study diet induced NAFLD in obesity^15^. The *Ldlr−/−.*Leiden substrain develops severe NASH/fibrosis in conjunction with pronounced dyslipidemia and IR. This model recapitulates metabolomics and transcriptomics profiles of NASH patients^16,17^. Moreover, *Ldlr−/−.*Leiden mice develop NASH and fibrosis without cholesterol supplementation, allowing the study of natural dietary fats and oils in a translational way.

On the basis that EVOO intake may contribute to an alleviation of the fatty liver grade in patients treated with a hypocaloric diet^18^ and can attenuate mild stages of NAFLD in C57BL/6J mice^13^, we tested whether EVOO-based HFDs may prevent development of IR and more severe stages of NAFLD using *Ldlr − / − .* Leiden mice. We found that EVOOs with both regular and high content of phenolic compounds consistently improved BW and insulin sensitivity but maintained liver inflammation and fibrosis.

## Results

### EVOOs-based diets reduced BW gain and altered plasma cholesterol

**Figure 1:**
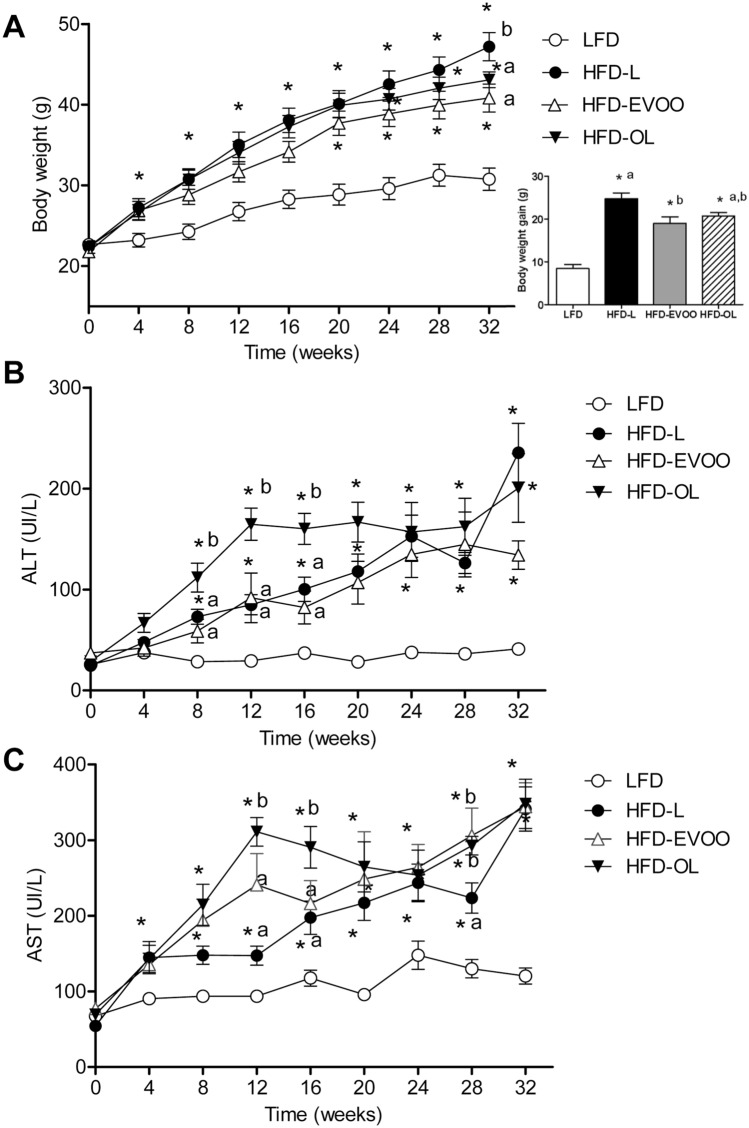
Body weight and transaminases (ALT and AST) values along the nutritional intervention study. (**A**) Left panel: BW during the 32 weeks of the nutritional intervention study (n = 17); Right panel: BW gain between the first and the last week of the study. (**B**) ALT and (**C**) AST values measured during the study (n = 7). Values are the means ± SEM. *p < 0.05 LFD vs HFDs groups (HFD-L, HFD-EVOO and HFD-OL); different letters indicate significant differences among HFD groups (*p* < 0.05).

Figure 1A (left panel) shows the BW development of the groups during the 32 weeks of nutritional intervention. All groups had a comparable BW at start. Mice treated with the different HFDs showed a similar BW increase throughout the study with significant differences to LFD which remained low. There were no significant differences in absolute BW between the olive oil treatment groups and the HFD-L group. However, calculation of total BW gain over the course of the study (Fig. 1A, right panel) revealed that the HFD-EVOO group gained significantly less weight than the HFD-L group (*p* < 0.05), and the same effect was observed for the HFD-OL group (*p* < 0.05). Feed efficiency (body mass gain in grams per kilocalories consumed) was significantly greater in HFD-L group (*p < 0.05) when compared to the rest of the groups (Supplementary Table S1). Analyses of transaminases (Fig. 1B,C) showed that both alanine aminotransferase (ALT) and aspartate aminotransferase (AST) plasma levels significantly increased (*p < 0.05) after week 8 in all HFD groups when compared to the LFD group.

**Figure 2:**
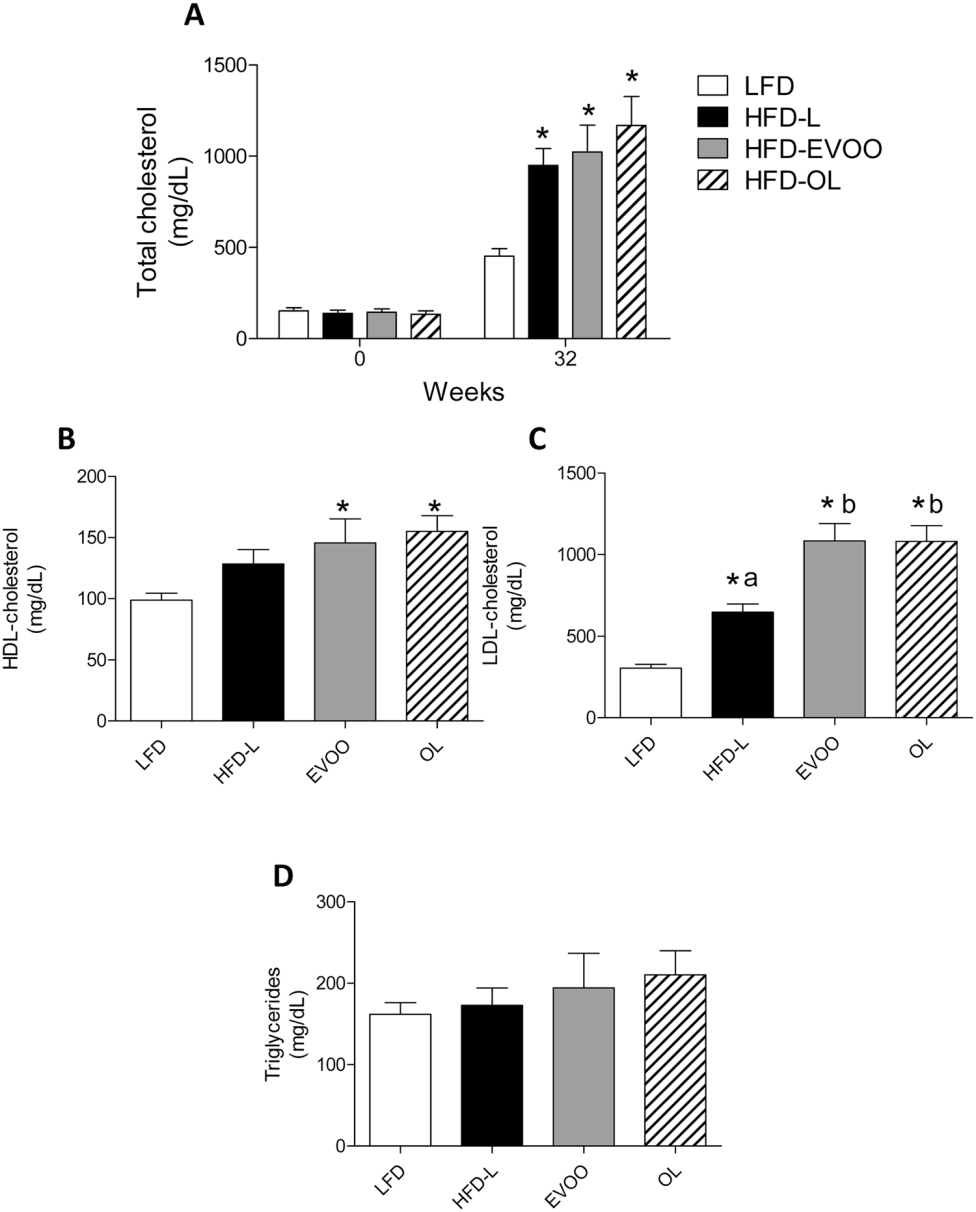
Effects of different diets on the lipid profile after 32 weeks of nutritional intervention study. (**A**) Plasma total cholesterol values at 0 and 32 weeks, (**B**) HDL-cholesterol, (**C**) LDL-cholesterol and (**D**) triglycerides values, at the end of nutritional intervention study. N = 12. Values are the means ± SEM. *p < 0.05 LFD vs HFDs groups (HFD-L, HFD-EVOO and HFD-OL); different letters indicate significant differences among HFD groups (*p* < 0.05).

Figure 2A shows that, at the end of the nutritional intervention study, all HFDs mice significantly (*p < 0.05) increased their total cholesterol levels (TC) compared to that of the LFD group. Regarding HDL-cholesterol (Fig. 2B), only the two HFD-EVOOs groups showed a significant increase of HDL (*p < 0.05 vs LFD). In the case of LDL-cholesterol, all HFDs groups showed a significant (*p < 0.05) increase relative to the LFD group, as expected in this model. Of note, both olive oil treated groups had significantly (*p* < 0.05) higher LDL-cholesterol levels than the HFD-L group (Fig. 2C). No significant differences were observed in plasma triglyceride (TG) levels (Fig. 2D).

### EVOOs based diets improved insulin sensitivity and reduced adipose tissue inflammation

**Figure 3:**
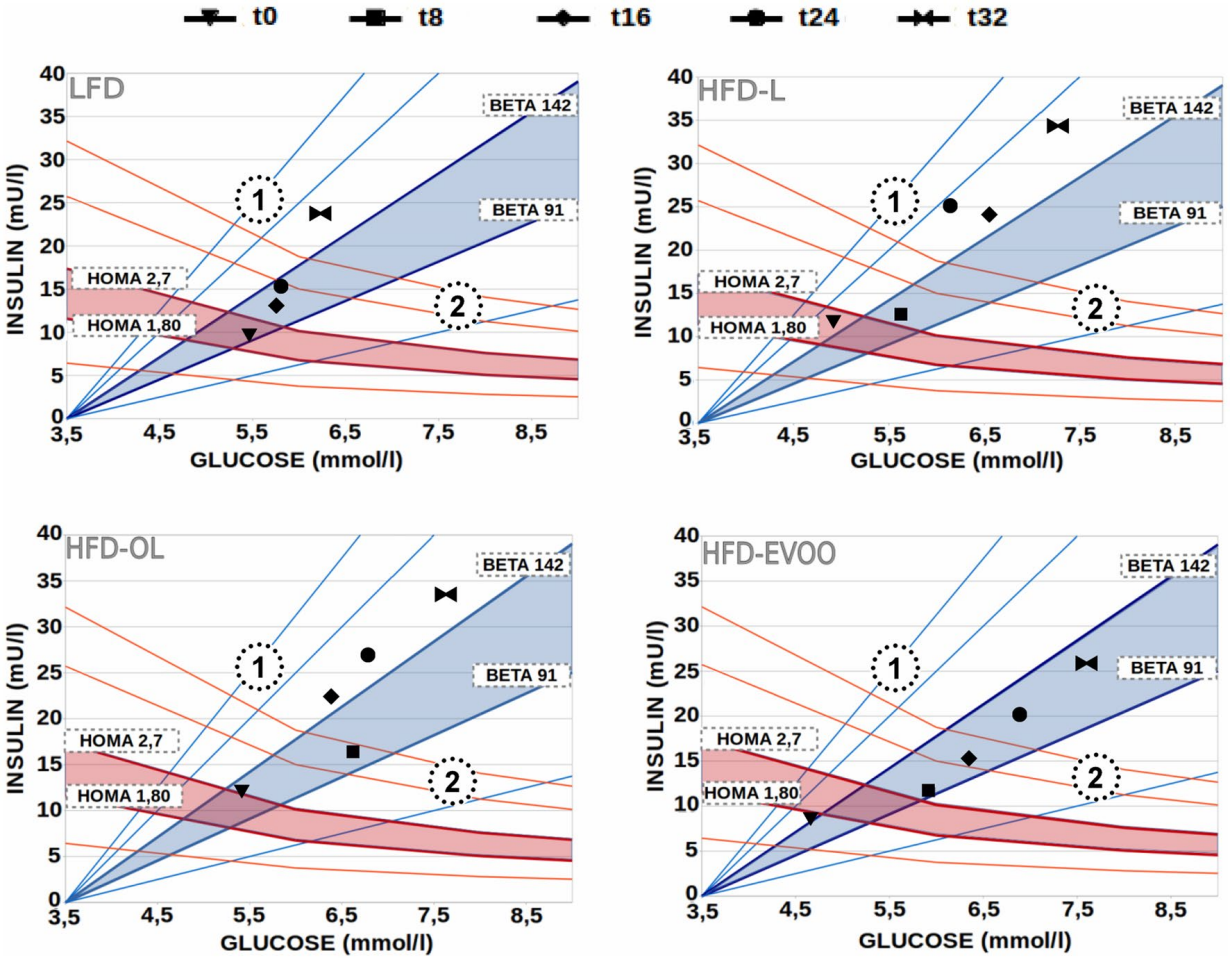
Fasting insulinemia vs glycemia with iso-HOMA-IR and iso-HOMA-%B curves at various timepoints (t) of the study (0, 8, 16, 24, and 32). Blue shadow zones represent the normality range for HOMA-%B (BETA 142 and BETA 91). Red shadow zones represent the normality range for HOMA-IR (HOMA 2.7 and HOMA 1.80). The normality ranges were established using fasting insulinemia and glycemia values at week 0 (t0). The average value and the upper value of the confidence interval for the HOMA-IR index were used, as well as the average value and the lower value of the confidence interval for the HOMA-%B. Area 1 reflects increased HOMA-IR and HOMA-%B. Area 2 reflects increased HOMA-IR and decreased HOMA-%B. The fasting insulinemia and glycemia values represent the means of 10 mice for each nutritional intervention.

Figure 3 shows fasting insulinemia *versus* glycemia with iso- homeostasis model assessment of IR (HOMA-IR) and iso-homeostasis model assessment of β-cell dysfunction (HOMA-%B) curves at various weeks of the study. Mice of the LFD group exhibited normal plasma glucose values but had elevated fasting insulin levels pointing to IR. At the end of the study mice of this group were in area 1, indicating that their pancreatic β-cells released more insulin. At that time, the mice were 44–47 weeks old suggesting that the observed IR on LFD is at least partially caused by aging. On the other hand, mice of the HFD-L and HFD-OL groups were in area 1 after only 16 weeks of HFDs feeding. By contrast, mice of the HFD-EVOO group were not observed in area 1 throughout the entire nutritional intervention and mice remained within the blue shadow zone, representing the normality range for HOMA-%B. OGTT, glucose and insulin concentrations, along the tests, were significantly (*p < 0.05) higher for HFD-L and HFD-OL groups when compared with LFD and HFD-EVOO groups (Supplementary Figure S1, panels A and B). For IPGTT, blood glucose values were significantly (*p < 0.05) higher in HFD groups compared to LFD. In the case of insulin, HFD-EVOO plasma values were significantly (*p < 0.05) higher than LFD but lower (p < 0.05) than HFD-L and HFD-OL (Supplementary Figure S1, panels C and D. This finding implies that the β-cells of EVOO treated mice exhibited a lower compensatory increase in insulin release. In addition, concerning liver IR, no significant differences were observed in basal hepatic insulin-resistance index between LDL and EVOO groups (1.0 ± 0.30 and 1.37 ± 0.21, respectively). However, HFD-L and OL groups showed significantly (*p < 0.05) higher values of this index (3.13 ± 0.47 and 2.96 ± 0.37, respectively).

The histological analysis of white adipose tissue showed that HFD-L group resulted in a significant increase (***p < 0.001) in adipocyte size relative to LFD group. The increase was significantly lower in both HFD-EVOO and HFD-OL groups (**p < 0.01) compared to LFD group. Moreover, significant differences (*p* < 0.001) were observed between both olive oil groups and HFD-L group (Supplementary Figure S2, panel B). Finally, HFD-L group showed significant increases in crown-like structures (CLS) versus LFD group (**p < 0.01) and both olive oil groups (*p* < 0.05). The lowest amonut of CLS, among the HFD groups, was observed in HFD-EVOO group (Supplementary Figure S2, panel C).

### EVOO-based diets induced steatosis, inflammation and fibrosis comparable to HFD-L reference diet

**Figure 4:**
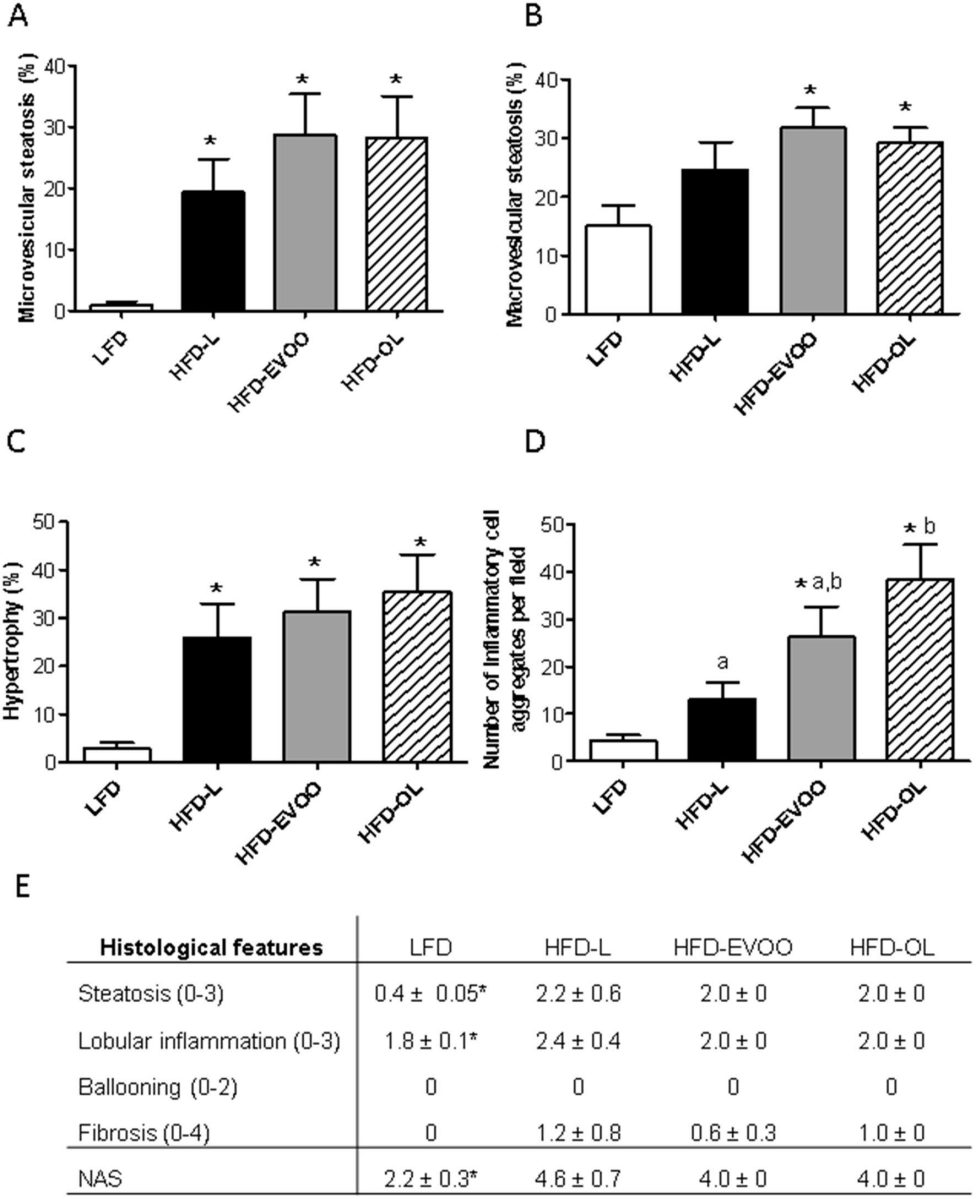
Diagnosis diagram for NAFLD after 32 weeks of nutritional intervention study. Values reported for steatosis based on the percentage of the total area affected: (**A**) Microvesicular, (**B**) Macrovesicular and (**C**) Hypertrophy. (**D**) Number of inflammatory foci per field at 100 × magnification. (**E**) Values reported for steatosis, lobular inflammation, hepatocyte balloon degeneration, fibrosis and NAS (total score) according to the NASH CRN Scoring system. Values represent the means ± SEM (n = 9). *p < 0.05 LFD *vs* HFDs groups; different letters denote significant differences between HFD groups (*p* < 0.05).

At the end of the study, liver histology was analyzed using a general NAFLD scoring system for rodent models. All HFDs treated groups showed a significant increase (*p < 0.05) in the percentage of the cross-sectional liver area displaying microvesicular steatosis (Fig. 4A) and hypertrophy (Fig. 4C) when compared to LFD group. Of note both olive oil groups showed a significant increase (*p < 0.05) in the percentage of macrovesicular steatosis (*p < 0.05) (Fig. 4B) while HFD-L only had an insignificant stimulating effect. Altogether, there was no statistical difference between HFD-L and EVOO groups regarding steatosis development. The number of inflammatory cell aggregates stained with macrophage antibody-2 (MOMA-2), was significantly increased with both EVOOs relative to LFD (*p* < 0.05), and this effect was even more pronounced in HFD-OL (significantly higher than HFD-EVOO; Fig. 4D). Moreover, at the end of the study, the NAFLD Activity (NAS) score in the LFD group was significantly lower (*p < 0.05; 2.2 ± 0.3; n = 5) than HFD groups (Fig. 4E). No significant differences were observed between HFD groups (4.0 ± 0.0 in the EVOO and OL groups, and 4.6 ± 0.70 in HFD-L group; n= 5) (Fig. 4E). No fibrosis was observed in the LFD group, and the HFD groups showed a comparable fibrosis development (Fig. 4E).

**Figure 5:**
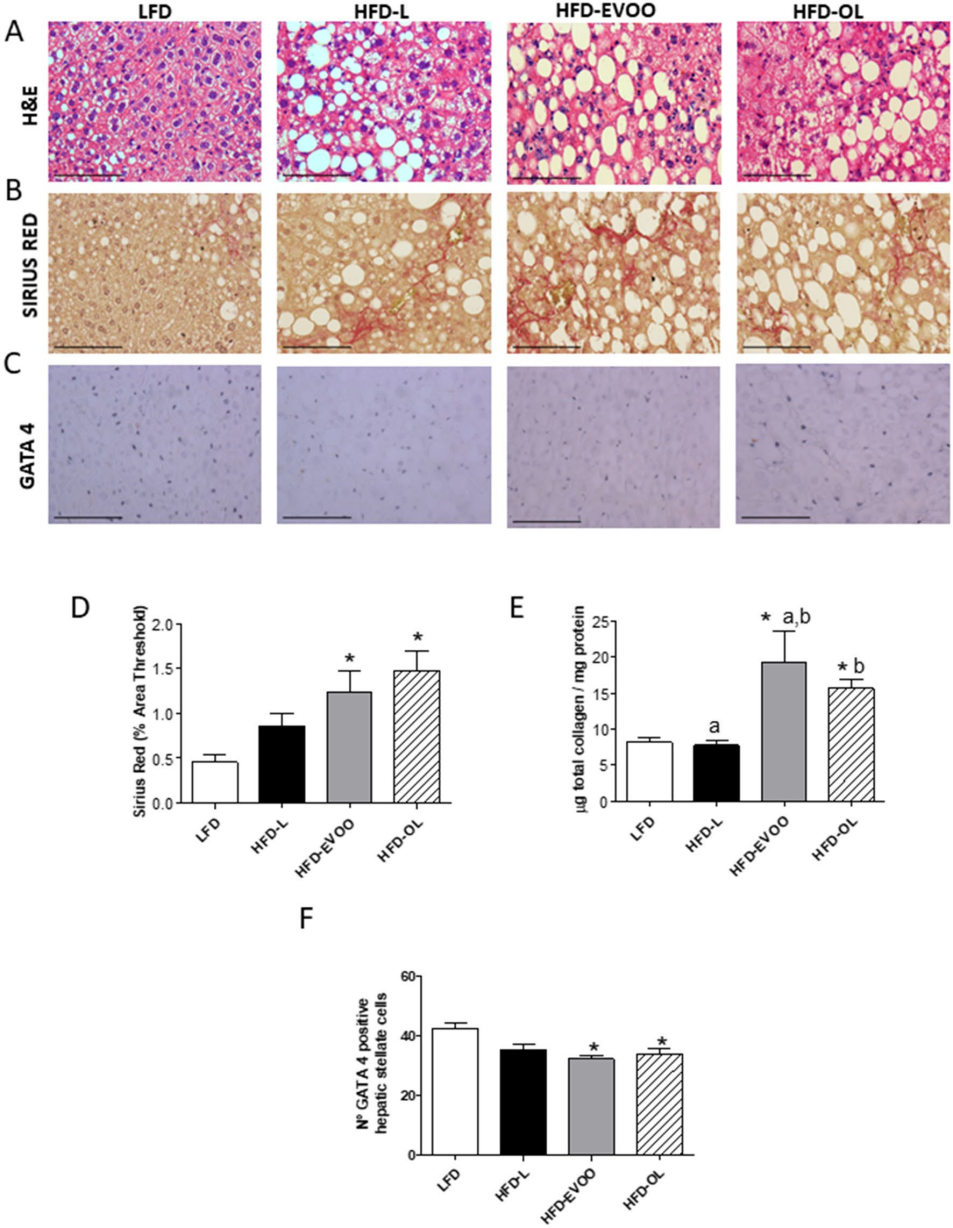
Liver stains after 32 weeks of nutritional intervention study. (**A**) H&E staining (40 ×). (**B**) Sirius Red staining (40 ×). (**C**) GATA 4 staining (40×). (**D**) Quantification of hepatic fibrosis by Sirius Red. (**E**) Quantification of total liver collagen (µg /mg liver protein) in liver homogenates. (**F**) Quantification of positive HSC per field. Values represent the means ± SEM (n = 10). *p < 0.05 LFD *vs* HFDs groups; different letters indicate significant differences between HFD groups (*p* < 0.05). Scale bars = 100 µm.

Hematoxylin–eosin staining of livers showed an increase in fat deposition in all HFDs treated groups compared to LFD (Fig. 5A). Moreover, both olive oil treated groups showed a significant (*p < 0.05) increase in the percentage of the Sirius Red positive area compared to LFD, indicating an increase in collagen deposition (Fig. 5B,D). In line with this, in liver homogenates of both EVOOs, higher amounts of biochemically-determined total collagen were found (*p < 0.05) (Fig. 5E). Figure 5C,F show that livers of both olive oil groups contained less (*p < 0.05) GATA binding protein 4 (GATA4) positive hepatic stellate cells (HSC), indicating a profibrotic state.

### Molecular signatures of NASH are induced by in both EVOOs containing diets

**Figure 6:**
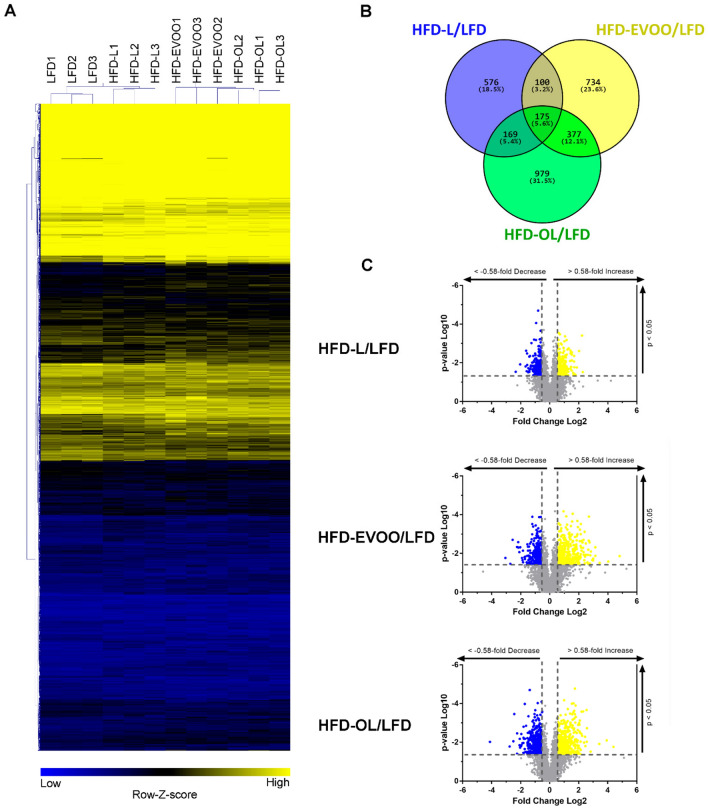
Hierarchical clustering, Venn diagram and scatter plots of differential expression profiles of LFD, HFD-L, HFD-EVOO and HFD-OL liver genes. (**A**) Hierarchical clustering of differential expression profiles of LFD, HFD-L, HFD-EVOO and HFD-OL genes, performed using Euclidean distance according to expression pattern with the MeV software. Scaled expression value, expressed as the row Z-score, is plotted in yellow-blue color scale, with yellow indicating high expression and blue indicating low expression. (**B**) Venn diagram showing the number and percentage of DEGs (up-regulated and down-regulated) shared by HFD-L, HFD-EVOO and/or HFD-OL relative to LFD. (**C**) Scatter plot showing the fold change and statistical significance of genes when comparing HFD-L, HFD-EVOO or HFD-OL relative to LFD. The yellow and blue dots represent up- and down-regulated genes, respectively, with statistical significance. The grey dots represent the genes without significant differential expression.

To explore the possible relationship between the above-mentioned changes in biochemical parameters and histopathological features with modifications in liver gene expression, we next analyzed the transcriptome in livers collected at the end of study using microarrays gene expression. We compared expression profiles of LFD, HFD-L, HFD-EVOO and HFD-OL as follows: First, hierarchical clustering was used to display the differentially expressed genes (rows) and different type of diets (columns) using half-square Euclidean distance as a similarity measure. Based on the hierarchical clustering of gene expression data, we identified distinct clusters with a distinct gene expression profile (Fig. 6A). Subsequent cluster analysis showed that HFDs differed from LFD (Fig. 6B). Mice consuming HFD-OL showed the highest amount of liver transcript changes (979) compared to LFD. Also, HFD-EVOO showed a large (734) amount of differentially expressed genes (DEG). In contrast, HFD-L mice showed the lowest amount of DEG (576) relative to LFD. Some DEG were common among the different groups, but only 175 DEG were shared among all conditions (Fig. 6B). This indicates (a) that the two olive oils differed markedly from each other despite their similar lipid composition and (b) that lard-based HFD and olive oil-based HFD evoked very different gene expression changes. The genes that exhibited significant up and down expression, when comparing HFD-L, HFD-EVOO or HFD-OL relative to LFD were evaluated by volcano plot filtering (Fig. 6C).

To gain insight in the specific effects of the various HFDs, we performed a DAVID analysis to identify potentially enriched biological processes using gene ontology (GO) terms. GO enrichment analysis revealed changes (up and downregulation) of several metabolism related DEG (see Supplementary Fig. S1). Interestingly, the metabolism-related DEG that were downregulated by both olive oils were genes involved in lipid-, cholesterol- and fatty acid-metabolism, as well as, lipid homeostasis (see Supplementary Fig. S3, middle and low panels).

**Figure 7:**
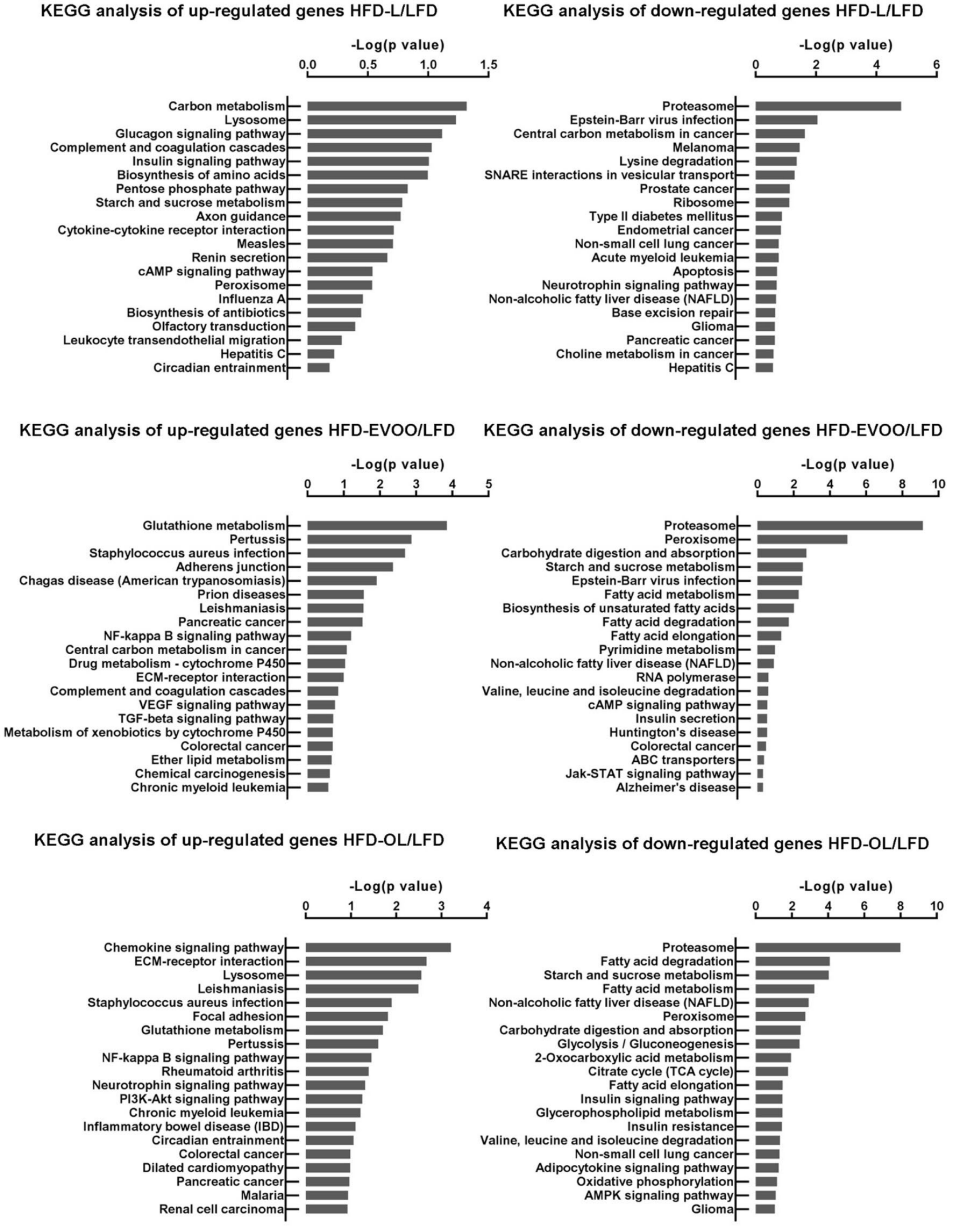
Predicted Biological Process GO terms identified using upregulated and downregulated DEG, in HDL, HDL-EVOO and/or HFD-OL relative to LFD, after 32 weeks of nutritional intervention study. DEGs were processed with DAVID tools v6.8. The p value was used to determine the significance of enrichment or overrepresentation of terms for each annotation. The ranking score was obtained using enrichment − log10 (p value) from the predicted target genes. The x-axis represents score and the y-axis represents the top enriched signaling pathways. The top 20 KEGG terms are listed as derived from the DAVID bioinformatics tool.

Besides, Kyoto Encyclopedia of Genes and Genomes (KEGG) enriched analysis of DEG shared by HFD-L, HFD-EVOO and HFD-OL reveals the “Top-20-Results up- and down-regulated” GO-term enrichment analysis, of the predominantly involved biological processes and the concomitant further in-depth delineation of the involved biological process and molecular functions (Fig. 7). Based on the findings shown in Fig. 7 (middle panel), it appears that glutathione metabolism genes are among the top-10 up-regulated GO-terms in the liver of both olive oil treated groups and differentially affected compared to LFD. This may suggest an effect of olive oils on redox homeostasis. To better characterize this effect, activity of glutathione peroxidase 1 (GPx) and glutathione reductase (GR) enzymes were measured in liver homogenates. Supplementary Figure S4 shows that GPx activity was significantly reduced (**p < 0.01) in HFD-EVOO group compared to the other groups (panel A). No significant differences were observed regarding GR activity among the different groups (panel B). In addition, the hepatic nuclear factor-erythroid 2 p45-related factor 2 (*Nrf2*) gene expression was measured (Supplementary Figure S5) and all HFD groups showed significant reductions (**p < 0.01) compared to the LFD group. In the case of the HFD-OL group, the most enriched GO-terms involve genes that are related to chemokine signaling pathway (Fig. 7, lower panel), possibly providing a rationale for the observed increase in inflammatory aggregates. Hepatic tumor necrosis factor alpha (*Tnf*_*α*_) gene expression was significantly increased (**p < 0.01) in all HFD groups with relative to LFD and consistent with this HFD groups also showed significant elevations in plasma TNF_α_ levels (***p < 0.001) when compared to LFD mice (Supplementary Figure S6). More specifically TNF_α_ plasma levels were significantly higher in the olive oil groups (**p < 0.01) than in the HFD-L group. The liver gene expression of *Il-6* was higher in the HFD-EVOO group (**p < 0.01) compared to the other groups. No significant differences were observed among all dietetic groups regarding interleukin 10 (*Il-10*) liver gene expression (Supplementary Figure S7, panels A, B and C). In addition, the signaling pathway leading to NF-kappa B activation is among the top-10 significantly affected GO-terms, in both olive oil groups, when compared with LFD (Fig. 7, middle and lower panel). An analysis of microarray data showed several genes involved in inflammatory response, that were up-regulated, with a ≥ twofold change (p < 0.05) in HFD groups versus LFD group. In many cases, the increase was higher in olive oil groups, particularly in OL group (Supplementary Table S2). It is noteworthy to mention that the transcripts encoding for profibrotic cytokines Tgf-β and osteopontin were strongly induced by both olive oils. Particularly the latter showed an extremely pronounced induction specifically with both olive oils, i.e. log_2_ fold changes of 21.02 (HFD-EVOO) and 28.02 (HFD-OL). Finally, in both olive oil fed groups (Fig. 7, middle and lower panel), genes related to fatty acid metabolism are among the top-10 down-regulated genes, when compared to LFD, suggesting that fatty acid metabolism and processing is downregulated compared to LFD, which is consistent with the view that de novo fatty acid synthesis could be shut down under high fat feeding conditions.

**Figure 8:**
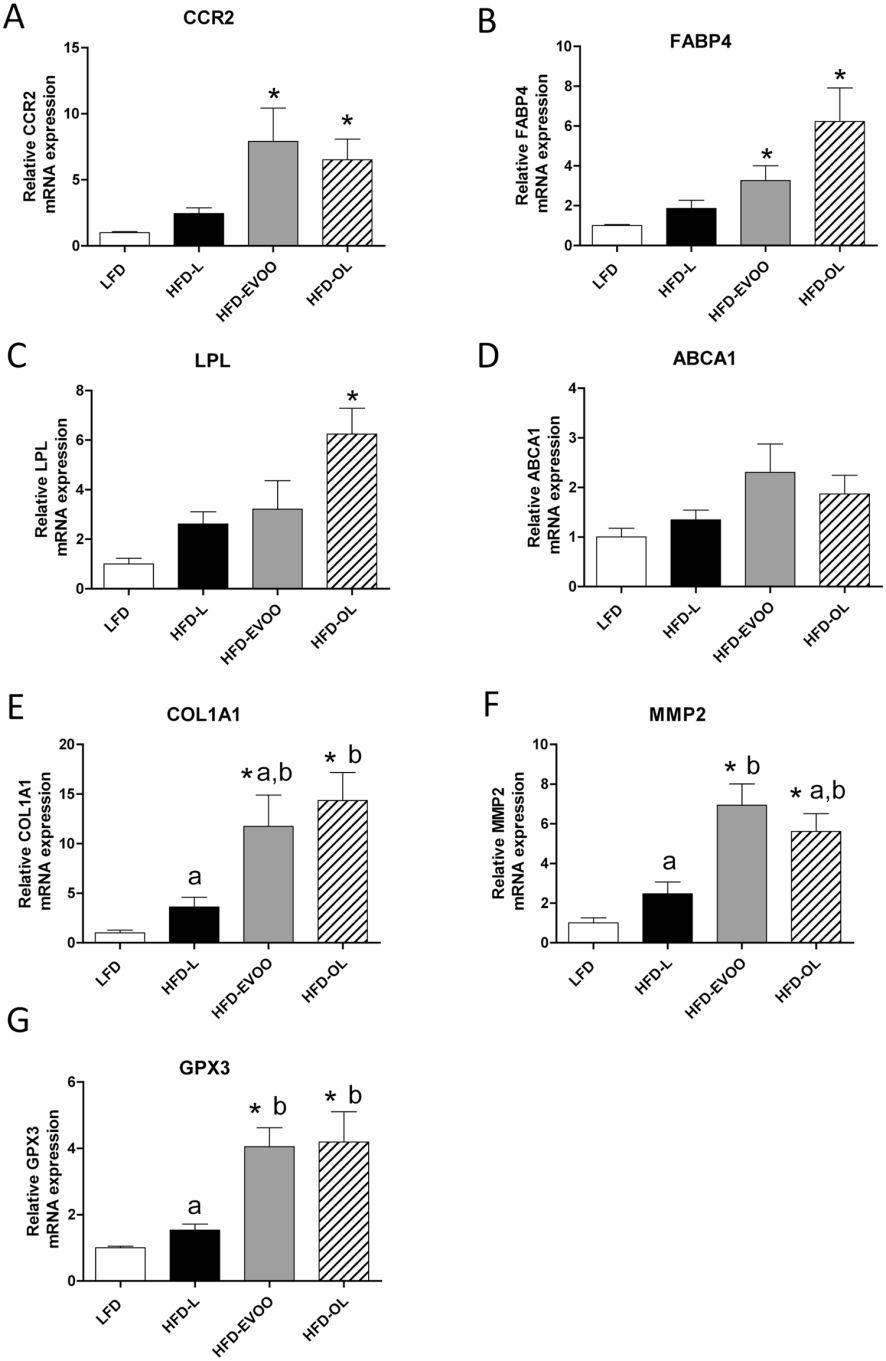
Hepatic mRNA expression of genes involved in inflammation, lipid metabolism, fibrosis and oxidative stress at 32 weeks of nutritional intervention. (**A**) Ccr2; (**B**) Fbpa4; (**C**) Lpl; (**D**) Abca1; (**E**) Col1a1; (**F**) Mmp2 and (**G**) Gxp3. Values are the means ± SEM (n = 3). *p < 0.05 vs LFD; different letters indicate significant differences between HFD groups (*p* < 0.05).

Accordingly to GO and KEGG enriched analysis, we studied the expression of genes related with the top-10 regulated GO terms. The analysis of the liver mRNAs encoding hepatic genes known to promote inflammation (C–C chemokine receptor type 2 (*Ccr2*) and fatty acid-binding protein 4 (*Fabp4*)) revealed that both olive oil treated groups had significantly (*p < 0.05) higher expression levels compared to LFD (Fig. 8A,B). In the case of genes related to lipid metabolism (lipoprotein lipase (*Lpl*) and ATP-binding cassette transporter 1 (*Abca1*)), HFD-OL mice showed a significant increase in *Lpl* (*p < 0.05), compared to LFD (Fig. 8C,D). Collagen type 1 alpha 1 chain (*Col1a1*) and matrix metalloproteinase-2 (*Mmp2*) gene expression were significantly increased (*p* < 0.05) in both olive oil treated groups, compared to the other groups (Fig. 8E,F) and a similar increase was found for glutathione peroxidase-3 (*Gpx3*) gene expression (Fig. 8G).

## Discussion

This study explores the effect of two EVOOs, with different content of phenolic components, to prevent HFD-induced NAFLD using a female mouse model (*Ldlr−/−.*Leiden) that develops severe forms of NASH and recapitulates features of the human pathology, when fed a HFD.

Importantly, we have used mature female mice because after reproductive age NAFLD prevalence is higher in women^19^. However, clinical and preclinical studies do not examine sex differences. In the case of preclinical studies, a possible explanation could be that females of the most frequently used genetic background for HFD-induced obesity analysis (C57BL/6J) gain less fat mass, develop a less pronounced metabolic phenotype and are more resistant to HFD-induced obesity and associated metabolic diseases^20^. A study investigating gender differences in young *Ldlr−/−.*Leiden mice (6 weeks old) using a lard-based HFD comparable to HFD-L showed that male mice were more susceptible to the detrimental effects of HFD^21^. Moreover, older female mice (more than 18 weeks) were predisposed to develop lobular inflammation independent of HFD feeding, suggesting a role for hormones and other inflammation-modulating factors. In the present study we used 12–17 weeks old female mice, and we observed pronounced steatosis and inflammation development. Together, these studies indicate (i) that mature female mice lost their ability to adapt to metabolic dietary stress and can develop steatosis similarly to male mice, and (ii) that the development of liver inflammation in the present study may, at least partly, mediated by hormonal and other sex-dependent factors^22^.

Herein, we examined the effects of the three HFDs on BW gain and liver damage, using plasma transaminase levels as readout. In a study using male C57BL/6 mice pretreated with L-HFD, an intervention with EVOO-based HFDs resulted in a maintenance of an obese phenotype, whereas L-HFD became slightly more obese^13^. However, another study using regular 12 weeks old *Ldlr−/−* mice^23^ showed that EVOO-based HFDs induced about the same BW gain than a lard-based HFD. The effect of EVOOs on BW remains controversial and might be dependent on both gender and other constituents of the diet^24^. In this study, EVOOs mice showed reduced BW gain compared to lard-treated mice, in line with those studies reporting BW-attenuating effects^13^.

As expected, feeding *Ldlr−/−.*Leiden mice with HFDs significantly elevated total cholesterol and LDL-cholesterol, indicative of dyslipidemia and consistent with NAFLD patients that display decreased liver LDL receptor expression and increased plasma LDL levels^25^. In line with our observations, Depner et al.^23^ also observed a significant increase in total plasma cholesterol levels in Ldlr*−/−* mice fed with HF-high cholesterol diet containing olive oil, when compared with mice fed a lard-based HFD.

Liver steatosis is associated with IR and IR is pivotal for the progression of NAFLD^26^. It is unclear whether IR of the liver, adipose tissue or skeletal muscle contributes most to NAFLD. *Ldlr−/−.*Leiden mice fed a HFD-L, for 16 weeks developed IR and their pancreatic β-cells release more insulin to normalize plasma glucose values. Based on longitudinal clamp study in male C57BL/6 mice fed a lard-based HFD, white adipose tissue-IR develops first (after about 12 weeks) and liver-IR requires 24 weeks of lard HFD feeding^27^. This suggests that the observed protective effects of EVOOs on IR, after 16 weeks of HFD feeding are likely mediated via beneficial effects on adipose tissue consistent with the effects reported by others and us^28^.

In this study, mice fed EVOO were not in area 1 of the Fig. 3, indicating less IR and the absence of a hypersecretory insulin response. A recent report showed that HFD-EVOO diets significantly improved the glycemic response and increased glucose sensitivity in male C57Bl/6J mice^29^. The observation that EVOO-HFD fed Ldlr*−/−*.Leiden mice developed lower IR than HFD-L and OL-groups could be due to the fact that these mice are suffering from a less severe condition of NAFLD which does not manifest histologically but on molecular level (e.g. lower GPx activity indicating less oxidative damage in conjunction with higher *Nrf2* expression) or because adipose tissue was less inflamed compared to the rest of the groups. We found no evidence for differences in systemic inflammatory mediators (including proinflammatory adipokines and cytokines)^30^ which could have constituted another mechanism that contributes to liver IR. In all, these data suggest that NAFLD can become worse on histological level in the presence of a mild liver IR which is consistent with retrospective human studies showing that a substantial portion (> 50%) of NAFLD patients does not suffer from IR^31^.

We observed that HFD groups developed more fibrosis compared to the LFD group, and treatment with EVOOs did not prevent the development of fibrosis. Delgado et al*.* showed that a reduction in liver *Gata4* expression can activate HSCs leading to chronic fibrosis^32^. We observed a decrease in *Gata4* positive HSC, indicating that EVOOs based HFDs induce fibrosis through an activation of HSC which is in concurrence with a significant increase of inflammation. The observed pronounced stimulation of osteopontin, an activator of HSCs^33^, by EVOO and OL may also have contributed to the observed effect on liver fibrosis.

EVOOs, particularly those rich in phenolic compounds have notable amounts of polyphenols that can exert anti-oxidant effects^13,28^. Dietary polyphenols have been shown to exert hormetic effects, i.e. they display protective effects at low doses and increase the generation of ROS when administered at high doses^34^. In fact, antioxidant balance is very important to prevent ROS generation and oxidative stress in fatty liver disease^35^. As the dietary EVOO content of our diets was high, it is possible that both EVOO groups received too high levels of dietary polyphenols resulting in the rather unexpected increase in hepatic inflammation and liver fibrosis. It is known that the transcription factor Nrf2 regulates the adaptive response to oxidative stress by inducing the expression of antioxidant genes and preventing NAFLD progression. Consistent with this, we observed a decrease in liver Nrf2 gene expression in HFD groups which may contribute to NAFLD^36^ and only the EVOO group showed significant *Nrf2* elevations indicating diminished oxidative stress in this group (consistent with aforementioned effects on GPx). The OL group did not show such effects and similarly to our study, detrimental effect was observed for chocolate preparations enriched with high amounts of polyphenols, where high chronic polyphenol consumption resulted in the aggravation of atherosclerosis^37^.

It has been demonstrated that dietary fat types reflect liver lipid composition and influences the progression of NAFLD by altering the liver gene expression profile^13^. Thus, we have analyzed the transcriptome of liver *Ldlr−/−.*Leiden mice consuming the different diets. The cluster analysis of microarrays showed a big difference in gene expression among HFDs, even between EVOO HFDs. Mice consuming HFD-OL, thus highest phenolic intake, showed the largest amount of DEG indicating that the phenolic components of EVOO are responsible for the observed gene expression changes, and potentially also for the histological outcomes related to these changes. EVOO fed mice showed a downregulation of genes involved in lipid-, cholesterol- and fatty acid metabolism, as well as, lipid homeostasis suggesting a shut down of lipid metabolism and/or lipid processing. This may cause an imbalance of liver lipid homeostasis, contributing to steatosis, as observed by histology. The most up-regulated genes, in both EVOO fed groups are those related to glutathione metabolism, indicating an effect of the oils on redox balance. Liver ROS overproduction may serve as a signaling mechanism to induce the expression of antioxidant genes to counteract the oxidative stress^38^. This could be happening in livers from mice fed EVOO-based HFDs. This suggestion is further confirmed in the case of EVOO group were a significant reduction of GPx activity indicates a reduced compensatory mechanism, suggesting that EVOO mice experience less liver damage because their ALT levels was also lower than those of HFD-L controls.

Cytokine plasma levels and liver gene expression corroborate the higher inflammatory state observed in HFD groups, particularly olive oil groups. KEGG analysis showed that genes related with chemokine and NF-kappa B signaling pathways are among the top-10 up-regulated pathways, particularly in the first position in HFD-OL. Marra and Tacke^39^ suggested that chemokines were directly involved in liver fibrogenesis. Moreover, NF-kappa B is involved in the transcription of multiple genes mediating liver inflammation, fibrosis and hepatocellular carcinoma^40^. For instance, NFkB controls the expression of *Ccr2* which promotes inflammatory cell recruitment in fatty liver and worsens NASH development^41^. Besides the role of NF-kB pathway, microarray analysis showed the participation of other genes (*Lcn2*, *Anxa2* or *Spp1*) related with inflammatory response, in the case of HFD groups. Finally, in EVOO fed mice, genes related to fatty acid metabolism are among the top-10 down-regulated genes (the top-5 down-regulated genes in HFD-OL fed mice). Because the lipid composition of EVOO and OL is comparable, this suggests that the phenolic components affect lipid metabolism genes. This confirms that there exists an imbalance of liver lipid homeostasis, which may increase liver steatosis in EVOO groups.

FABPs are a group of molecules that coordinate inflammatory and metabolic responses in cells. Few studies have assessed the involvement of hepatic *Fabp4* expression in NAFLD. Greco et al. have described *Fabp4* as being upregulated in subjects with high liver fat content^42^. The expression of *Fabp4* and *Fabp5* in the liver were correlated with hepatic fatty infiltration in NAFLD patients^43^. In the case of lipid metabolism, it is known that in obesity, the impaired ability to up-regulate LPL by insulin exacerbates hepatic postprandial lipid load, thus causing hepatic steatosis. Therefore, LPL regulation is a crucial component in hepatic lipid homeostasis. It has been shown, that as human NAFLD progresses, *Lpl* expression is elevated, specifically in HSCs^44^. *Col1a1* and *Mmp2* are genes involved in fibrosis processes. COL1A1 has been described as a potential biomarker of NAFLD/NASH, as its expression is increased in liver of patients with NAFLD^45^. In addition, it has been demonstrated in a model of Col1a1r/r mice that degradation of collagen-I is critical to hepatocyte regeneration and loss of activated HSC during spontaneous recovery from liver fibrosis^46^. In the case of MMP-2, it is produced by HSCs and plays an important role in liver fibrogenesis. In a model of obese Zucker rats with NAFLD, it was found a positive correlation between ROS production, HSC activation and an increase in the activity of MMP-2^47^. In addition, it has been found that ROS released from hepatocytes regulates MMP-2 expression in HSCs, mainly through NF-κB signaling pathway^48^. Finally, altered redox signaling due to generation of ROS is thought to play a pivotal role in NASH development^49^ and the observed increase in fibrosis upon EVOO treatment may be due to high ROS generation caused by chronic high polyphenol treatment. In a dietary murine model of NASH, in which gene families involved in oxidative stress were analyzed in liver tissue, it was shown that members of the GPx family, mainly GPx3 and GPx6, where the most significantly upregulated genes^50^. In our case, the observed increased expression of GPx3 suggests a compensatory mechanism that is triggered by enhanced ROS formation in the livers of mice fed EVOOs containing HFDs.

In conclusion, treatment of female *Ldlr − / − .*Leiden mice with extra virgin olive oil-based HFDs results in an improvement of weight gain, insulin sensitivity, and adipose tissue inflammation in absence of attenuating effects on liver steatosis, inflammation and fibrosis. This rather unexpected finding may be explained by a fundamental difference in the plasticity between these organs: while the adipose tissue can respond with hyperplasia and expand, the liver is anatomically restricted with a limited storage capacity so that even lipids that are beneficial in nature may cause physical damage to cells when present in excess amounts. Another possible explanation is related to the intake of relatively high amounts of phenolic compounds, which may, due to hormesis, cause an increase of hepatic ROS production. The polyphenols doses ingested by mice treated with EVOO and OL, when extrapolated to humans, are comparable to the dietary intake of extra virgin olive oil of people in the highest tertile of olive oil consumption in the PREDIMED Study^51^. Different from a normal human EVOO consumption pattern, the mice of this study consumed EVOOs (and their polyphenolic components) chronically at every feeding moment. It is possible that this chronic consumption causes effects that would not have been observed when the same dose of polyphenolic compounds was consumed during one feeding moment. Potential proposed mechanisms contributing to the observed chronic EVOO effects are the upregulation of genes involved in liver inflammation, fibrosis and oxidative stress, as well as the downregulation of genes critical for liver lipid homeostasis. Future long-term studies that examine the role of dietary oils that have high content of antioxidants are warranted.

## Methods

### Animals diets and experimental design

Female *Ldlr−/−.*Leiden mice (n = 68, aged 12–17 weeks at the start of the experiment) were bred and characterized in collaboration with TNO (The Netherlands Organisation for Applied Scientific Research, Leiden, The Netherlands). All procedures with animals were approved by the Institutional Animal Care Committee of CABIMER (permission number 06-10-14-138) and performed according to the Spanish law on animal use RD 53/2013 and the European Community policy for Experimental Animal Studies (Directive 2010/63/EU). In addition, the study was carried out in compliance with the ARRIVE guidelines (https://arriveguidelines.org). All mice were fed with food and water ad libitum throughout the 32-week research period. To determine water (21.4 ± 4.6 ml/cage/day) and food intake (21.3 ± 4.3 g/cage/day), four mice of each group were placed, for one week, in the TSE Phenomaster monitoring system (TSE Systems GmbH, Bad Homburg, Germany). No significant differences were observed in water and food intake among dietetic experimental groups. BW was recorded once per week throughout the study period. Mice were randomly divided into four groups at the start of the study. One group was fed a standard chow (low-fat diet (LFD); n = 17; 4% kcals fat, 14.3% kcals protein, 48% kcals carbohydrate; 2.9 kcal/g; 2014 Teklad Global, Harlan, Madrid, Spain). The other three groups were fed isocaloric HFDs (45% kcals fat). The HFD was prepared with the 2014 Teklad chow modified with different types of fats being the rest of macronutrients the same: HFD-L (41% kcals fat from lard, 11% kcals protein, 37% kcals carbohydrate; 5.9 kcal/g; n = 17); HFD-EVOO with a natural content of phenolic compounds (41% kcals fat from EVOO; n = 17), or HFD-OL based on EVOO richer in phenolic compounds (41% kcals fat from EVOO richer in phenolic compounds; n = 17). The HFD-EVOO group received 2.92 mg of polyphenols/kg of mouse body weight/day. The HFD-OL group received 6.08 mg of polyphenols/kg of mouse body weight/day. Both olive oils used for the study were of the Picual variety. Characterization of EVOOs and compositions of the experimental diets are indicated in Jurado-Ruiz et al.^29^. The concentration of total phenolic compounds in the EVOO and OL were 171 and 353 PPM, respectively. The phenolic composition of the EVOOs used is provided in Supplementary Table S3. Composition of the experimental diets is shown in Supplementary Table S4.

### Plasma biochemical analysis

Blood was collected from the tail vein of fasted overnight animals. Blood glucose was measured using an automatic glucometer (Accu-Chek Aviva, Roche Diagnostic, Indianapolis, IN, USA). TG, TC, HDL, LDL, AST and ALT were measured using a Cobas Integra 400 (Roche Diagnostics, Indianapolis, IN, USA). Insulin were measured using an ELISA kit from Mercodia (Mercodia AB, Uppsala, Sweden). IR was calculated according to Matthews et al.^52^.

### Liver histological evaluation

At the end of the study, liver sections, from fasted overnight mice, were stained with hematoxylin and eosin (HE), Oil Red O or Sirius-red. Livers were scored blindly by a certified pathologist according to the NAFLD scoring methods of Liang et al.^53^. In addition, histology alterations were evaluated by using the NASH CRN Scoring System. In Sirius-red staining, the area of fibrosis was evaluated by direct pixel counting on binary images captured under × 10 magnification, and the average was calculated from 10 images per liver. Fibrosis was also assessed using Masson’s trichrome. Liver tissue sections were subjected to dewaxing, hydration and thermal induction antigen retrieval. Slices were blocked and incubated with anti-GATA4 (1:100, Santa Cruz Biotechnology, Dallas, TX, USA) and anti-MOMA-2 antibodies (1:100, Abcam, Cambridge, UK) at 4ºC overnight. For GATA4, the slices were incubated with the peroxidase substrate diaminobenzidine using the Vectastain Elite ABC kit (Vector Laboratories, Burlingame, CA, USA). MOMA-2 slices were incubated with an Alexa Fluor 647 secondary antibody (1:300, Abcam, Cambridge, UK). All slices were observed under light and fluorescence microscopy (Leica, Wetzlar, Germany). Nuclei were counterstained with DAPI. Image analysis was performed using IMAGE J software (National Institutes of Health, Bethesda, MD, USA) and normalized with the tissue area observed.

### Liver hydroxyproline assay

Snap frozen liver tissues were subjected to acid hydrolysis for 20 h at 95 °C. Hydroxyproline, a component of collagen, was measured in the hydrolysate using a total collagen assay relative to a collagen standard per the manufacturer´s instructions (Quickzyme, Leiden, The Netherlands).

### Microarray

Total RNA was isolated using the RNeasy Mini Kit (Qiagen, Venlo, The Netherlands) from three LFD and HFDs liver mice (25 mg) and 100 ng was used to obtain the gene expression profile of each sample. Biotinylated cRNA (2,3 µg) prepared from total RNA was hybridised on an Affymetrix Clariom S Mouse Array (Affymetrix, Santa Clara, CA, USA). Microarrays were scanned using GeneChip Scanner 3000 7G (Affymetrix, Santa Clara, CA, USA) and were processed with AFFYMETRIX COMMAND CONSOLE 4.0 Software (AGCC) (Affymetrix, Santa Clara, CA, USA) to extract raw data (CEL file). Microarray expression data were analysed by the TRANSCRIPTOME ANALYSIS CONSOLE (TAC) Software 3.0 from Affymetrix (Affymetrix, Santa Clara, CA, USA). Expression values were normalized by SST (Signal Space Transformation)-RMA method^54^ and differential gene expression (DGE) analysis was then performed using One-Way (single factor) ANOVA. Genes were considered significantly upregulated or downregulated when their expression values were fold change (linear) > 2 and ANOVA *p* value < 0.05. Gene Ontology (GO) and functional annotation were performed using the Database for Annotation, Visualization and Integrated Discovery (DAVID Tools v6.8)^55^. Enrichment of Biological Process GO terms were considered as statistically significant when *p* value < 0.05. Dendrograms and hierarchical clustering heat maps were generated using the open-source data analysis MULTIPLE EXPERIMENT VIEWER (MeV) software package version 4.0. To identify the related pathways, we used the Kyoto Encyclopedia of Genes and Genomes (KEGG) pathway database^56^. Microarray gene expression data were confirmed by quantitative real-time q-PCR.

### RNA isolation and real time q-PCR (RT-qPCR)

Total RNA was extracted from snap frozen livers using the RNeasy Mini Kit (Qiagen, Hilden, Germany). RNA quantity was determined using a NanoDrop spectrophotometer (Thermo Scientific, Wilmington, DE, USA) and the quality was assessed using an Agilent 2100 Bioanalyzer (Agilent Technologies, Santa 6 Clara, CA, USA). Total RNA was reverse transcribed to complementary DNA using the NZY First Strand cDNA Synthesis Kit (Nzytech, Lisbon, Portugal). Quantitative PCR was performed in triplicate for each sample using TaqMan Gene Expression Assays on a 7500 Fast Real Time PCR System (Applied Biosystems, Foster City, CA, USA). The Taqman probes used for expression analysis of target genes are detailed in Supplementary Table S5. Results are expressed as relative gene expression normalized to the expression levels of the housekeeping gene *Gapdh*.

### Statistical analysis

The results were presented as means ± SEM. The normality of distribution was checked using the Shapiro–Wilk test. Statistical analysis was performed using one-way analysis of variance (ANOVA), followed by Dunnett´s test, for comparison of LFD group with HFDs groups or Scheffe's test to detect significant differences among HFD groups. For non-normally distributed continuous data, statistical analysis was performed using the Kruskal–Wallis test and post hoc tests using Bonferroni-corrected Mann–Whitney U test or Dunn test for multiple comparisons. *P* values < 0.05 were considered statistically significant. GraphPad Prism 8.0 (La Jolla, CA, USA) was used for drawing the graphics and statistical analysis.

## Supplementary Information

Supplementary Information

## Data Availability

The data sets generated during and/or analysed during the current study are available from the corresponding author or reasonable request.
