# Supplementary material for: Extra virgin olive oil improved body weight and insulin sensitivity in high fat diet-induced obese LDLr−/−.Leiden mice without attenuation of steatohepatitis
Source: Sci Rep. 2021 Apr 15;11:8250. doi: 10.1038/s41598-021-87761-3 (PMC8050103; doi:10.1038/s41598-021-87761-3)
Supplement: Supplementary file 1 — Supplementary Information [file 41598_2021_87761_MOESM1_ESM.docx]

Extra virgin olive oil improved body weight and insulin sensitivity in high fat diet-induced obese LDLr-/-.Leiden mice without attenuation of steatohepatitis

Leticia Álvarez-Amor^1,2^, Amparo Luque Sierra^1^, Antonio Cárdenas^1,2^, Lucía López-Bermudo^1^, Javier López-Beas^1^, Eloísa Andújar^1^, Mónica Pérez-Alegre^1^, Rocío Gallego-Durán^3,4^, Lourdes M. Varela^1^, Alejandro Martin-Montalvo^1^, Genoveva Berná^1,2^, Anabel Rojas^1,2^, Mª José Robles-Frías^5^, Abdelkrim Hmadcha^2^, Manuel Romero-Gómez^3,4^, Robert Kleemann^6,7*^, Franz Martín^1,2*^.

*^1^Andalusian Center of Molecular Biology and Regenerative Medicine-CABIMER, University Pablo Olavide-University of Seville-CSIC, Seville, Spain.*

*^2^Biomedical Research Network on Diabetes and Related Metabolic Diseases-CIBERDEM, Instituto de Salud Carlos III, Madrid, Spain.*

*^3^Hospital Universitario Virgen del Rocío de Sevilla, Instituto de Biomedicina de Sevilla, Universidad de Sevilla, Sevilla, Spain.*

*^4^Biomedical Research Network on Hepatic and Digestive Diseases-CIBEREHD, Instituto de Salud Carlos III, Madrid, Spain*.

*^5^Compared Pathology Unit, Hospital Universitario Virgen del Rocío-Intituto de Biomedicina de Sevilla Biobank Node, Andalucia Health Public System Biobank, Sevilla, Spain.*

*^6^Department of Metabolic Health Research, Netherlands Organisation for Applied Scientific Research-TNO, 2333 CK, Leiden, Netherlands.*

*^7^Department of Vascular Surgery, Leiden University Medical Centre, Leiden, 2333 ZA, Netherlands.*

**SUPPLEMENTARY INFORMATION**

**Methods**

Diet design and fabrication

The EVOOs were administered to the animals via the food. We used powdered diet to prepare the HFDs. Different fats (lard, EVOO and OL) were added and mixed with the powdered diet (217 g of fats/kg of mouse feed). To make pelleted forms of each of those diets 10% water was carefully mixed with the powdered diet. The resulting slurry then was pelleted (diameter 1.30 cm) using a laboratory pellet mill. Then, the pellets were air-dried at 29 ºC for 24h. All diets were prepared twice per month and stored at -20 ºC. Fresh diet was provided to mice every other day.

Phenols analysis

The phenolic fraction was isolated by solid phase extraction and analysed by reverse phase HPLC using a diode array UV detector according to Mateos *et al* (2001) [1]. The quantification of phenolic compounds other than flavones and ferulic acid was carried out at 280 nm using p-hydroxyphenylacetic acid as an internal standard, while flavones (luteolin and apigenin) and ferulic acid were quantified at 335 nm using o-coumaric acid as an internal standard. The results were expressed in mg kg^−1^.

Feed efficiency calculation

The feed efficiency was calculated as indicated by Fraulob JC *et al* [2] with the following equation [(weight gained in g/kcal consumed) x 100], for the different animal groups.

Histological analysis of visceral white adipose tissue

Adipose tissue cryosections (10µm) were stained with hematoxylin and eosin. Adipocyte size and crown-like structure (CLS) numbers were measured under 10x magnification in 20 random fields per mouse. All slices were observed under light and fluorescence microscopy (Leica, Wetzlar, Germany). Image analysis was performed using IMAGE J software (National Institutes of Health, Bethesda, MD, USA).

Oral and intraperitoneal glucose tolerance tests (OGTT and IPGTT)

Both tests were performed at the end of the nutritional intervention. For OGTT and IPGTT mice were fasted 6h or overnight, respectively and then gavaged orally or injected intraperitoneally with glucose (2 g/kg body weight) and blood samples were collected at 0, 15, 30, 60 and 120 min. To measure glucose-stimulated insulin secretion, at same time points, blood samples were collected, and plasma was isolated and stored at -80 ºC for the insulin analysis. Blood glucose was measured using an automatic glucometer and insulin was assayed by ELISA using the Mercodia kit (Mercodia AB, Uppsala, Sweden).

Basal hepatic insulin-resistance index

This index is computed as the product of the total area under the curve (AUC) for glucose and insulin during the first 30 min of the OGTT (glucose_0-30_[AUC] × insulin_0-30_[AUC]) [3]. The area under the curve (AUC) was calculated using the trapezoid rule [4].

Glutathione peroxidase (GPx) and glutathione reductase (GR) activity assays

Liver samples were prepared using 50 mg of homogenized liver tissue. The activity liver GPx was assessed using a colorimetric assay (ab102530, Abcam, Cambridge, UK) according to a glutathione reductase-coupled method. The principle of the test is the reduction of the hydroperoxide molecule by GPx, which gives rise to the oxidized form of glutathione. The GR assay (ab83461, Abcam, Cambridge, UK) is based on measuring spectrophotometrically the resulting chromophore (TNB). GPx and GR activity assays of liver were performed by following the manufacturer’s instructions provided with each kit.

Measurement of TNFα plasma levels

Plasma TNFα was measured using murine cytokine‑specific ELISA kit (BMS607-3, Thermo Fisher Scientific, Inc., Barcelona, Spain) according to the manufacturer's protocol. The concentration of TNFα was measured as marker of inflammatory reaction using a microplate reader at 450 nm.

Liver *Nrf2* gene expression

The target gene *Nrf2* was assessed using forward and reverse designed primers (F: 5’-CGA GAT ATA CGC AGG AGA GGT AAG A-3’, oligo ID 201201B017H10; R: 5’-gct cga caa tgt tct cca gct t-3’, oligo ID 201201B017A1, Metabion, Planegg, Germany). According to the manufacturer’s instructions, qPCR reaction was conducted in 20 µL of reaction buffer containing 10 µL Taq polymerase (TB Green Premix Ex Taq; Takara, Shiga, Japan), 0.4 µL ROX II, 0.8 µL of 10 µM forward and reverse primers, 1 µL of 1 µg cDNA, and 7.8 µL water. The RNA expression levels were normalized to the level of GAPDH expression. Quantitative PCR was performed in triplicate for each sample using a 7500 Fast Real Time PCR System (Applied Biosystems, Foster City, CA, USA).

**Supplementary Table S1:** Feed efficiency in different nutritional intervention groups.

| Feed efficiency (%) | Nutritional Intervention groups | | | |
| --- | --- | --- | --- | --- |
|  | LFD | HFD-L | HFD-EVOO | HFD-OL |
|  | 57.0 ±4.9 | 80.3±5.2* | 58.7±3.1 | 66.1±4.2 |

Feed efficiency is measured as body mass gain in grams per kilocalories consumed. Values are means ± SEM (n= 17/group). **p*<0.05 *vs* rest of the groups

**Supplementary Table S2:** Changes in liver genes expression involved in inflammatory response, after 32 weeks of nutritional intervention. Genes with a change of log2-fold or more are shown, along with the corresponding p values, are indicated if higher than 2 and less than 0.05.

| Gene symbol | HFD-L | | EVOO | | OL | |
| --- | --- | --- | --- | --- | --- | --- |
|  | Log2 fold change | p-value | Log2 fold change | p-value | Log2 fold change | p-value |
| *Ano6* | 2.06 | 0.025 | 3.67 | 0.003 | 3.58 | 0.0013 |
| *Anxa2* | 3.41 | 0.041 | 4.14 | 0.045 | 5.52 | 0.015 |
| *Axl* | 2.12 | 0.0004 | 2.47 | 0.010 | 2.88 | 0.0001 |
| *C3ar1* | 2.01 | 0.021 | 5.61 | 0.011 | 2.59 | 0.005 |
| *Ccr2* | 2.01 | 0.0007 | 3.75 | 0.011 | 2.3 | 0.03 |
| *Cd44* | 2.05 | 0.010 | 3.36 | 0.005 | 4.41 | 0.002 |
| *Cd74* | 2.03 | 0.049 | 7.67 | 0.0008 | 4.25 | 0.001 |
| *Clec12a* | 2.07 | 0.017 | 2.40 | 0.021 | 3.54 | 0.0078 |
| *Ctss* | 2.38 | 0.0019 | 4.51 | 0.005 | 3.36 | 0.003 |
| *Cybb* | 2.87 | 0.007 | 3.45 | 0.004 | 3.81 | 0.0001 |
| *Fcer1g* | 2.47 | 0.047 | 3.68 | 0.010 | 2.88 | 0.009 |
| *Gsn* | 2.56 | 0.026 | 6.68 | 0.002 | 4.69 | 0.004 |
| *Il1a* | 2.02 | 0.015 | 3.25 | 0.038 | 3.04 | 0.049 |
| *Irak3* | 2.71 | 0.002 | 3.13 | 0.042 | 3.26 | 0.035 |
| *Lcn2* | 4.66 | 0.0003 | 5.43 | 0.005 | 8.82 | 0.01 |
| *Mmp2* | 2.13 | 0.032 | 5.02 | 0.008 | 5.91 | 0.009 |
| *Prkcd* | 2.07 | 0.028 | 2.34 | 0.025 | 2.09 | 0.026 |
| *Selplg* | 2.04 | 0.018 | 2.61 | 0.030 | 2.05 | 0.010 |
| *Sirpa* | 2.06 | 0.009 | 4.34 | 0.004 | 5.12 | 0.0002 |
| *Slc11a1* | 2.23 | 0.047 | 2.41 | 0.032 | 2.33 | 0.039 |
| *Spi1* | 2.07 | 0.043 | 3.12 | 0.008 | 4.23 | 0.003 |
| *Spp1* | 3.14 | 0.047 | 21.02 | 0.013 | 28.02 | 0.017 |
| *Tgb1* | 2.66 | 0.045 | 2.65 | 0.011 | 2.94 | 0.0003 |
| *Tlr7* | 2.10 | 0.023 | 4.22 | 0.016 | 4.46 | 0.016 |
| *Tnfaip3* | 2.18 | 0.035 | 5.08 | 0.011 | 3.92 | 0.016 |
| *Tyrobp* | 2.81 | 0.044 | 3.56 | 0.005 | 3.76 | 0.014 |

**Supplementary S3:** Mean concentration of sterols and phenols identified in the EVOOs used for this study (PPM).

|  | **EVOO** | **OL** |
| --- | --- | --- |
| **Phenols (PPM)** |  |  |
| Hydroxytyrosol (HTY) | 1.22 | 4.00 |
| Tyrosol (TY) | 6.01 | 15.29 |
| 1^st^ HTY derivative | 7.95 | 9.66 |
| 1^st^ TY derivative | 6.40 | 4.73 |
| 2^nd^ HTY derivative | 14.71 | 13.86 |
| 2^nd^ TY derivative | 68.07 | 33.10 |
| Vanillic acid | 0.78 | 2.09 |
| Vanillin | 0.21 | 0.35 |
| HTY Acetate | 1.89 | 8.48 |
| TY Acetate | 0.00 | 0.00 |
| Pinoresinol | 2.64 | 3.50 |
| Cinnamic acid | 0.00 | 0.00 |
| Acetoxypinoresinol | 1.69 | 3.46 |
| Ferulic acid | 58.79 | 253.56 |
| Total polyphenols | 171.99 | 353.60 |
| Total orto-phenols | 26.87 | 36.59 |
| Total secoiridoids | 97.12 | 61.35 |

**Supplementary Table S4:** Dietary composition of the experimental diets.

| **Diet (kcal %)** | **LFD** | **HFD** | **HFD-EVOO** | **HFD-OL** |
| --- | --- | --- | --- | --- |
| **Carbohydrate** | 48.0 | 37 | 37 | 37 |
| **Protein** | 14.3 | 11 | 11 | 11 |
| **Fat** | 4.0 | 45 | 45 | 45 |
| **% Saturated** | 0.6 | 18 | 5.1 | 5.1 |
| **% Monounsaturated** | 0.7 | 20 | 33.7 | 33.7 |
| **% Polyunsaturated** | 2.1 | 5.4 | 5.4 | 5.4 |
| **% from soybean oil** | 4.0 | 4.0 | 4.0 | 4.0 |
| **% from lard** | 0 | 41 | 0 | 0 |
| **% from EVOO** | 0 | 0 | 41 | 41 |
| **Energy (kcal/g)** | 2 | 4 | 4 | 4 |

All values in the table, with the exception of the fat source (soybean oil, lard or EVOO), represent the percentage of the total kcal in the respective diets derived from the indicated macronutrients. The values for the fat source represent the percentage of the total kcal from fat derived from the indicated source. The main carbohydrate and protein sources are wheat and corn.

**Supplementary Table S5:** Gene-specific oligonucleotides used for the real-time quantitative PCR analysis.

| **Gene** | **Gene Bank no.** | **TaqMan ID** |
| --- | --- | --- |
|  | **Mouse** |  |
| *Abca1* | NM_013454.3 | Mm00442646_m1 |
| *Ccr2* | NM_009915.2 | Mm00438270_m1 |
| *Col1a1* | NM_007742.3 | Mm00801666_g1 |
| *Fabp4* | NM_024406.2 | Mm00445878_m1 |
| *Gapdh* | NM_008084.3 | Mm99999915_g1 |
| *Gpx3* | NM_008161.3 | Mm00492427_m1 |
| *Il-10* | [NM_010548.2](http://www.ncbi.nlm.nih.gov/nuccore/NM_010548.2) | Mm01288386_m1 |
| *Lpl* | NM_008509.2 | Mm00434764_m1 |
| *Mmp2* | NM_008610.2 | Mm00439498_m1 |
| *Tnfα* | [NM_013693.3](http://www.ncbi.nlm.nih.gov/nuccore/NM_013693.3) | Mm00443258_m1 |

**
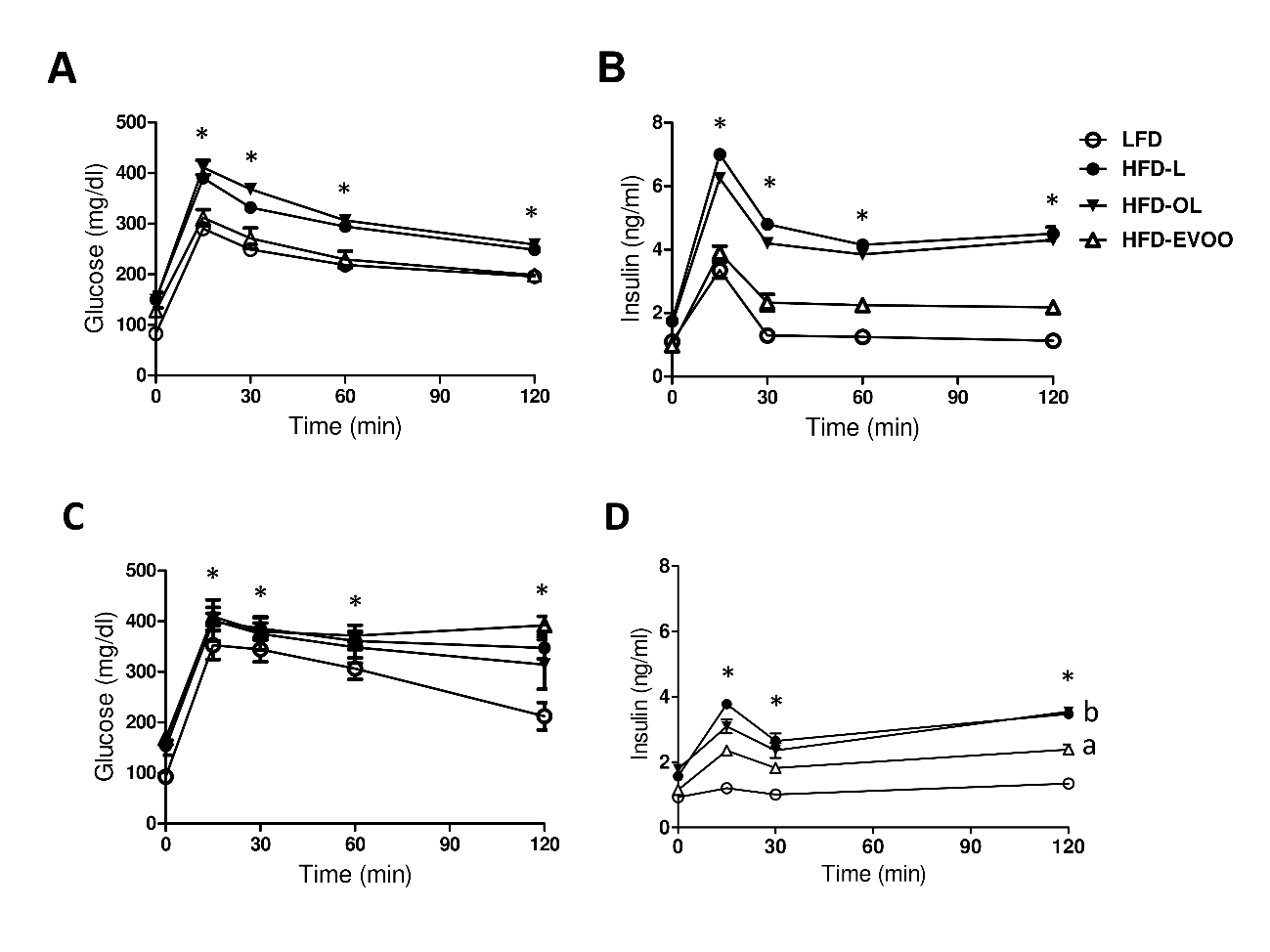
**

**Supplementary Figure S1. OGTT and IPGTT of different dietetic groups after 32 weeks of nutritional intervention.** (A) Blood glucose concentrations during the OGTT. (B) Insulin concentrations during the OGTT. (C) Blood glucose concentrations during IPGTT. (D) Insulin glucose concentrations during IPGTT. Values are means ± SEM (n= 6). *p< 0.05 vs LFD and HFD-EVOO; different letters indicate significant differences among HFD groups for all time points (p<0.05).

**
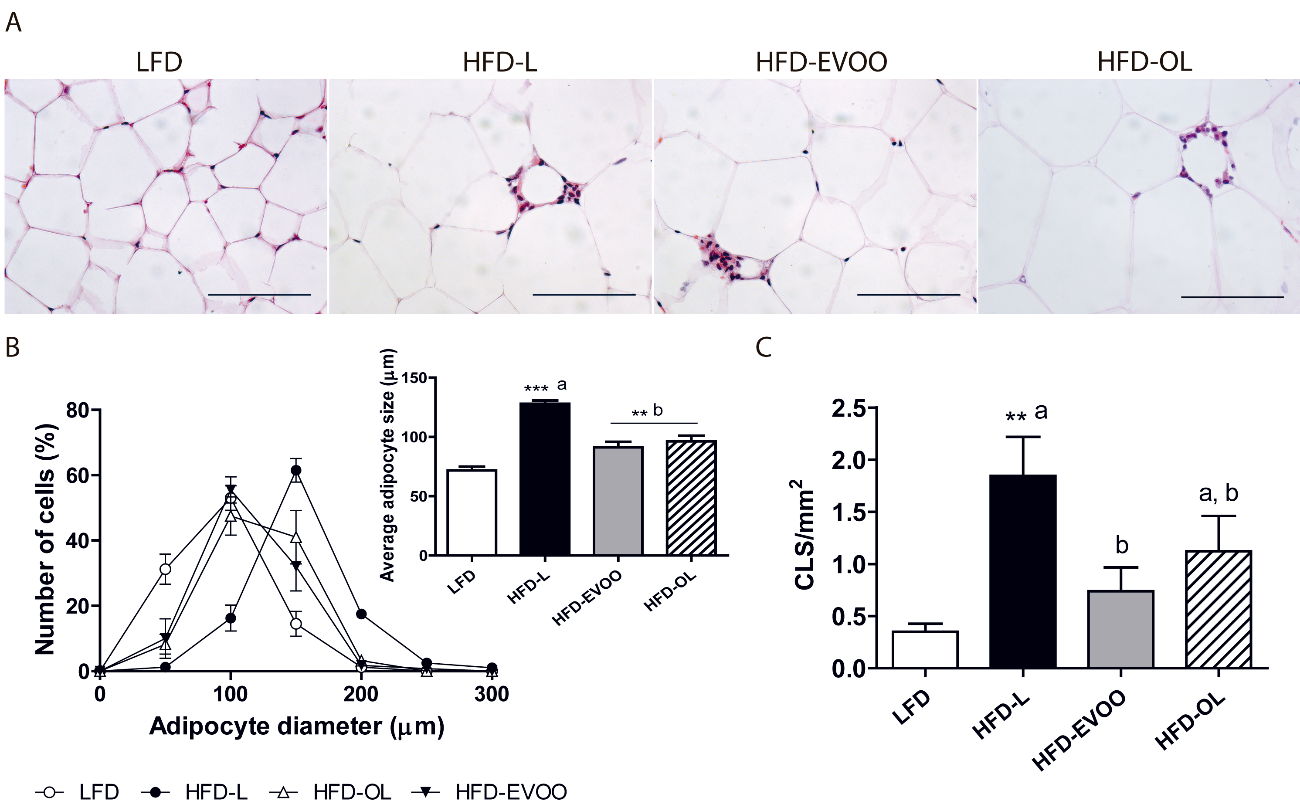
**

**Supplementary Figure S2. Histological analysis of** **Ldlr-/-.Leiden mice white visceral adipose tissue after 32 weeks of nutritional intervention.** (A) Representative images of H & E-stained adipose tissue (40x, scale bar = 100 µm). (B) Size distribution and average size of adipocytes. (C) Percentage of crown-like structures (CLS) present in the adipose tissue. Values are means ± SEM (n= 5). **p< 0.01/***p<0.001 vs LFD; different letters indicate significant differences among HFD groups (p<0.05).

**Supplementary Figure S3.** **Scatterplot of GO analysis from DEGs shared by HFD-L, HFD-EVOO and/or HFD-OL relative to LFD livers, showing the most enriched biological processes (enrichment score ≥ 1.0) after 32 weeks of nutritional intervention study.** DEGs for each comparison were processed with DAVID tools. GO scatterplots were generated using the open-source online tool REViGO. Bubble color indicates the p-value of GO terms (expressed as log10 p-value), where blue and green bubbles are GO terms with lower p-values than the orange and red bubbles. Bubble size indicates the frequency of the GO term in the underlying GO database.

**
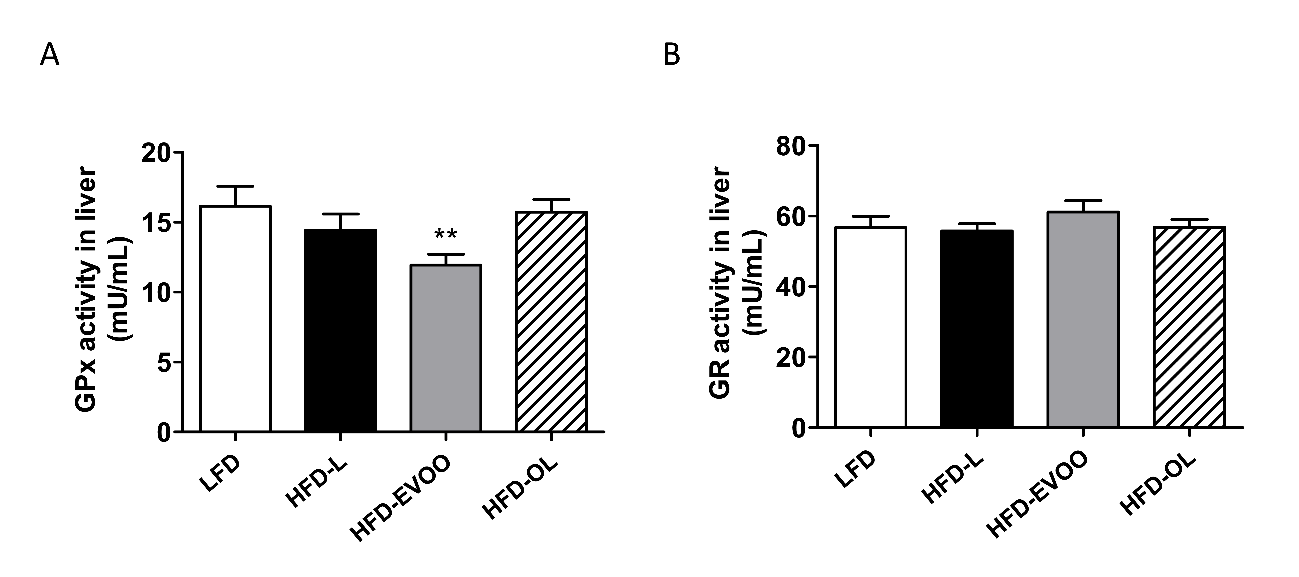
**

**Supplementary Figure S4. Glutathione liver enzymatic activity after 32 weeks of nutritional intervention.** (A) Glutathione peroxidase (GPx). (B) Glutathione reductase (GR). Values are means ± SEM (n=5).**p<0.01 vs rest of the groups.

**
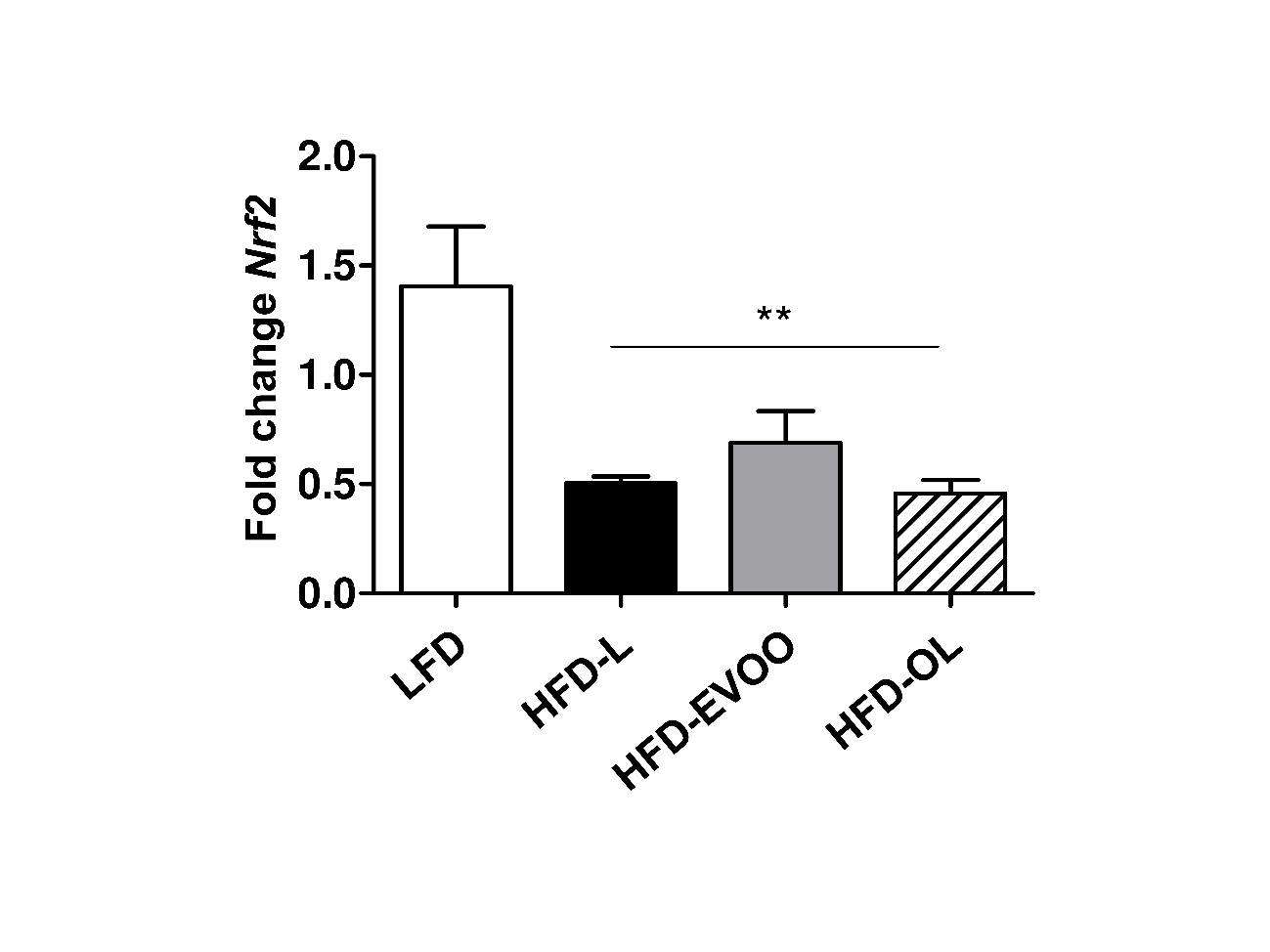
**

**Supplementary Figure S5. Hepatic mRNA Nrf2 expression at 32 weeks of nutritional intervention.**  Values are the means ± SEM (n = 5). **p<0.01 LFD vs HFD groups.

**
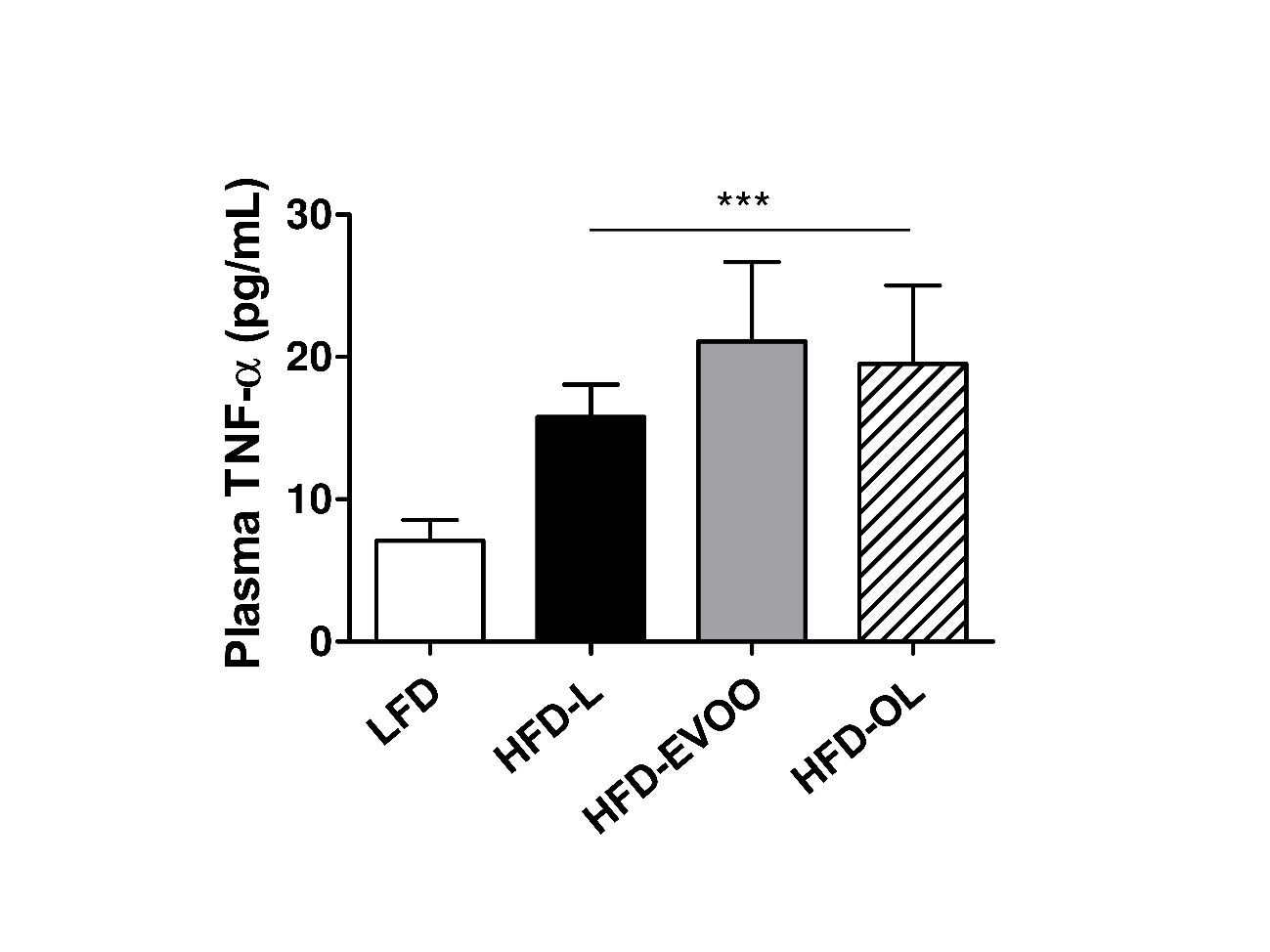
**

**Supplementary Figure S6.**  **Plasma TNFα levels of mice at 32 weeks after nutritional intervention.** Values are the means ± SEM (n = 8). ***p<0.001 LFD vs HFD groups.


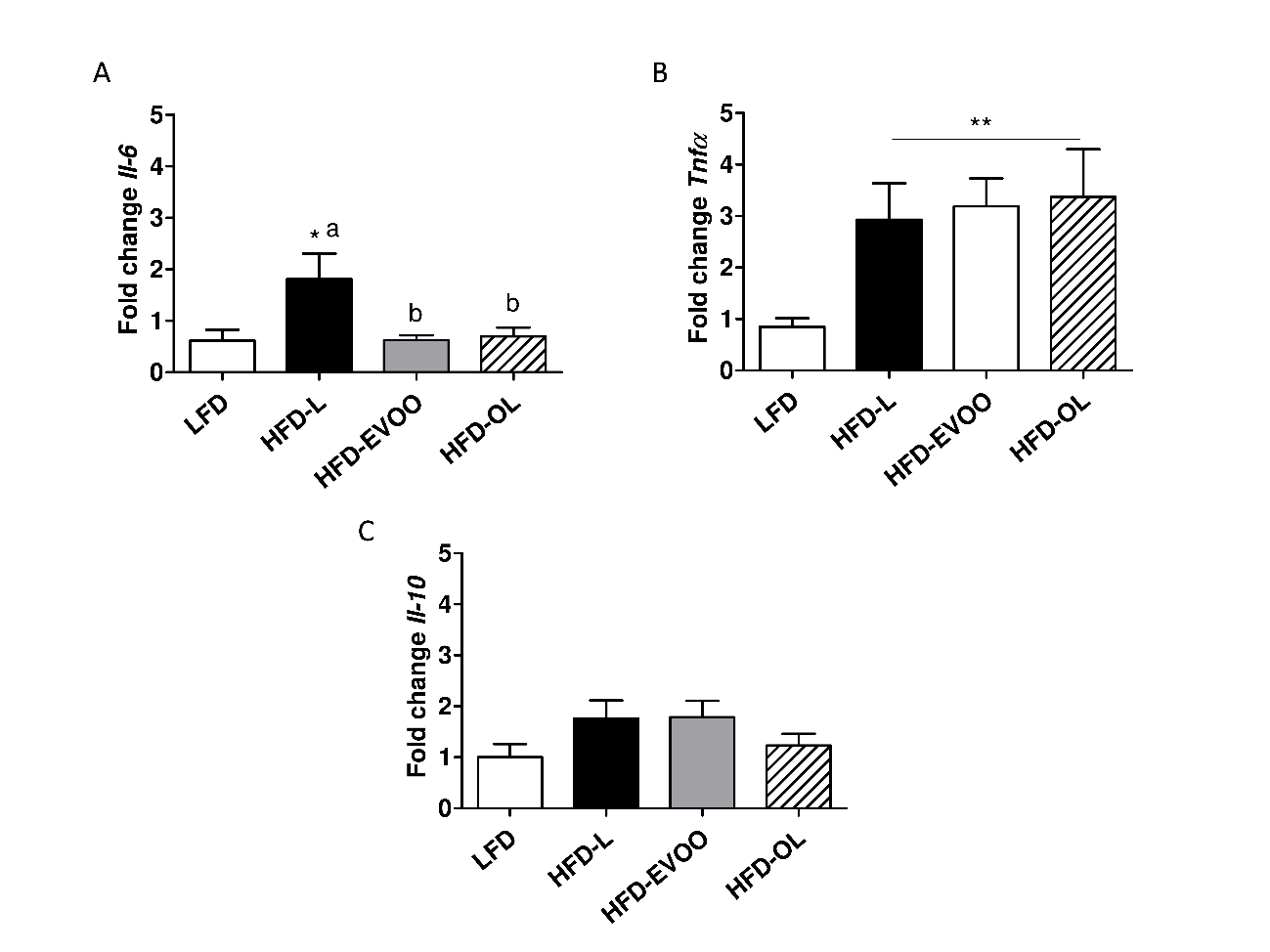


**Supplementary Figure S7. Hepatic mRNA Il-6, Tnfα and Il-10 expression at 32 weeks of nutritional intervention.** (A) Il-6; (B) Tnfα; (C) Il-10. Values are the means ± SEM (n = 6). *p<0.05 vs rest of the groups. **p<0.01 LFD vs HFD groups; different letters indicate significant differences between HFD groups (p<0.05).

**References.**

1. Mateos, R et al. Determination of phenols, flavones, and lignans in virgin olive oil by solid-phase extraction and high-performance liquid chromatography with diode array ultraviolet detection. J. Agric. Food Chem. **49,** 2185-2192 (2001).
2. Fraulob, J.C., Ogg-Diamantino, R., Fernandes-Santos, C., Barbosa-Aguila, M. & Mandarim-de-Lacerda, C.A. A mouse nodel of metabolic syndrome: insulin resistance, fatty liver and non-alcoholic fatty pancreas disease (NAFPD) in C57BL/6 mice fed a high fat diet. J. Clin. Biochem. Nutr. **46**, 212-223 (2010).
3. Gastaldelli, A. et al. Relationship between hepatic/visceral fat and hepatic insulin resistance in nondiabetic and type 2 diabetic subjects. Gastroenterology, **133**, 496-506 (2007).
4. Turner, R.C. *et al*. Applications of structural model of glucose-insulin relations to assess beta-cell function and insulin sensitivity. *Horm. Metab. Res.* **24,** 66-71 (1990).
